# Assessing mentoring: A scoping review of mentoring assessment tools in internal medicine between 1990 and 2019

**DOI:** 10.1371/journal.pone.0232511

**Published:** 2020-05-08

**Authors:** Yong Xiang Ng, Zachary Yong Keat Koh, Hong Wei Yap, Kuang Teck Tay, Xiu Hui Tan, Yun Ting Ong, Lorraine Hui En Tan, Annelissa Mien Chew Chin, Ying Pin Toh, Sushma Shivananda, Scott Compton, Stephen Mason, Ravindran Kanesvaran, Lalit Krishna

**Affiliations:** 1 Yong Loo Lin School of Medicine, National University of Singapore, Singapore, Singapore; 2 Division of Supportive and Palliative Care, National Cancer Centre Singapore, Singapore, Singapore; 3 Lee Kong Chian School of Medicine, Nanyang Technological University, Singapore, Singapore; 4 Medical Library, National University of Singapore Libraries, National University of Singapore, Singapore, Singapore; 5 Department of Family Medicine, National University Health System, Singapore, Singapore; 6 Education Department, Duke-NUS Medical School, Singapore, Singapore; 7 Cancer Research Centre, Marie Curie Palliative Care Institute, University of Liverpool, Liverpool, England, United Kingdom; 8 Division of Medical Oncology, National Cancer Centre Singapore, Singapore, Singapore; 9 Centre of Biomedical Ethics, National University of Singapore, Singapore, Singapore; 10 Division of Cancer Education, National Cancer Centre Singapore, Singapore, Singapore; 11 PalC, The Palliative Care Centre for Excellence in Research and Education, Singapore, Singapore; Faculty of Science, Ain Shams University (ASU), EGYPT

## Abstract

**Background:**

Mentoring’s success in enhancing a mentee’s professional and personal development, and a host organisations’ reputation has been called into question, amidst a lack of effective tools to evaluate mentoring relationships and guide oversight of mentoring programs. A scoping review is proposed to map available literature on mentoring assessment tools in Internal Medicine to guide design of new tools.

**Objective:**

The review aims to explore how novice mentoring is assessed in Internal Medicine, including the domains assessed, and the strengths and limitations of the assessment methods.

**Methods:**

Guided by Levac et al.’s framework for scoping reviews, 12 reviewers conducted independent literature reviews of assessment tools in novice mentoring in PubMed, Embase, Scopus, ERIC, Cochrane, GreyLit, Web of Science, Open Dissertations and British Education Index databases. A ‘split approach’ saw research members adopting either Braun and Clarke’s approach to thematic analysis or directed content analysis to independently evaluate the data and improve validity and objectivity of the findings.

**Results:**

9662 abstracts were identified, 187 full-text articles reviewed, and 54 full-text articles included. There was consensus on the themes and categories identified through the use of the split approach, which were the domains assessed and methods of assessment.

**Conclusion:**

Most tools fail to contend with mentoring’s evolving nature and provide mere snap shots of the mentoring process largely from the mentee’s perspective. The lack of holistic, longitudinal and validated assessments propagate fears that ethical issues in mentoring are poorly recognized and addressed. To this end, we forward a framework for the design of ‘fit for purpose’ multi-dimensional tools.

**Practice points:**

- Most tools focus on the mentee’s perspective, do not consider mentoring’s evolving nature and fail to consider mentoring holistically nor longitudinally
- A new tool capable of addressing these gaps must also consider inputs from all stakeholders and take a longitudinal perspective of mentoring

## Introduction

Mentoring in medicine helps shape a mentee’s professional identity and personal development, and enhances the career, progress and satisfaction of mentors and mentees [1, 2]. It also boosts the reputation of host organisations [3–19]. These successes rely on the development of personalised mentoring relationships, nurtured through personalised, appropriate, specific, timely, longitudinal, accessible and holistic mentoring support [20–26].

However, mentoring’s ability to provide consistent personalised support is suspect given the lack of robust assessments of mentoring processes that can detect problems and direct timely and appropriate support to the mentee and mentor [27, 28]. This gap raises concerns that ethical issues in mentoring which include the lack of mentoring support, the misappropriation of mentee’s work, bullying and inappropriate behaviour may also be overlooked [29–36]. Two recent reviews into the potential sources of ethical issues in mentoring in medicine and surgery found that mentoring assessment tools continue to intermix of mentoring with coaching, supervision, tutoring and role-modelling and conflate distinct mentoring practices such as novice, near-peer, peer, group, mosaic, network and e-mentoring and mistakenly [20, 37–41]. In addition Lee et al. (2019) [42] and Cheong et al. (2019) [43] found that prevailing assessments of mentoring processes are too reliant upon “*Cartesian reductionism and Newtonian principles of linearity*” [28] and fail to contend with mentoring’s longitudinal, competency based, evolving, adapting, entwined, goal-sensitive, context-specific, mentor-, mentee-, mentoring relationship and host organisation-dependent nature (henceforth mentoring’s nature) [44, 45]. These shortcomings compromise effective evaluations of mentoring processes and relationships and reiterate the need for urgent review of assessments of mentoring processes [42, 43].

### The need for this review

To address gaps in assessing mentoring tools, a scoping review is proposed to map “how is mentoring processes, support, relationships, outcomes and the oversight of the mentoring programs assessed?”. Acknowledging mentoring’s nature and recognizing that mentoring assessment tools need to be specific and contextualised for each particular form of mentoring, data form this review promises to inform design of a assessment framework that could act as a template for the construction of individualised mentoring tools [29–36].

## Methods

This scoping review seeks to map prevailing tools published in peer-reviewed and grey literature [46–52], identify knowledge gaps in the field, and set the basis for an assessment framework and a systematic review of mentoring assessment tools [53]. Mentoring’s context specific nature requires that this scoping review confine itself to mapping practice to a specific form of mentoring and a particular speciality. To this end, this review will focus on novice mentoring, the dominant form of mentoring in medical education [39, 54]. Novice mentoring is defined as *“a dynamic*, *context-dependent*, *goal-sensitive*, *mutually beneficial relationship between an experienced clinician and junior clinicians and/or undergraduates focused upon advancing the development of the mentee*” [55].

Levac et al. [48]’s refinement of Arksey and O’Malley [46]’s framework for scoping reviews was used to organise the methods and results of this review. This stage-wise framework is as follows:

### 1) Identifying the research question

The 12-member research team (henceforth the research team) discussed the research question with medical librarians from the Yong Loo Lin School of Medicine (YLLSoM) and the National Cancer Centre Singapore (NCCS) and sought advice from educational experts and clinicians at the NCCS, YLLSoM, the Palliative Care Institute Liverpool and Duke-NUS Medical School (henceforth the expert team).

Guided by the expert team, the research team determined the primary research question to be “**what tools are available to assess mentoring in novice mentoring in Internal Medicine?**” The secondary research questions were “**What domains are evaluated by available mentoring assessment tools?**”, “**When and how are these tools deployed?**” and **“Are prevailing tools to assess novice mentoring validated?”**.

Envisioning that the findings of this scoping review will guide design of mentoring tools; the comprehensiveness and feasibility of prevailing tools were also considered given the longitudinal nature of the mentoring process. This is outlined in Table 1 which also shows the PICOS format that was used to guide this study.

**Table 1:** PICOs, inclusion criteria and exclusion criteria applied to database search.

PICOs	Inclusion criteria	Exclusion criteria
**Population**	Undergraduate Medical students	Allied health specialties such as dietetics, nursing, psychology, chiropractic, midwifery, social work, psychology, Physiotherapy, Occupational therapy, Podiatry,
	Graduate Medical Student	
	Junior clinicians	
	Postgraduate Residents	
	Senior clinicians	
	Attendings	Non ACGME internal medicine medical specialties such as Clinical and Translational Science, Veterinary, Dentistry, Military medicine, Obstetrics and Gynaecology, Paediatrics, Anaesthesia, Pathology, Family Medicine, Surgery, Urology, Orthopaedics, Ophthalmology, Complementary medicine, Athletic medicine, Osteopathy, Radiation oncologist, Translational medicine
	Consultants	
**Intervention**	Method of mentoring assessments	
	Method of evaluating mentoring	
**Comparison**	Tools used in mentoring assessment	
	Tools used in mentoring evaluation	
**Outcome**	Type of tools used to evaluate mentoring	
	Target of assessment in mentoring
		Peer mentoring, near-peer mentoring, mentoring for leadership, mentoring patients or mentoring by patients
	Areas assessed in mentoring	
**Study Design**	All qualitative methodologies and quantitative designs (observation studies, randomised controlled trials, cohort studies, cross sectional studies, longitudinal studies and case studies)	
		Role modelling, coaching, supervision, advising, preceptorship

### 2) Identifying relevant studies

Given novice mentoring’s context specific nature, this review scrutinises accounts of novice mentoring in all subspecialties of Internal Medicine as defined by the Accreditation Council for Graduate Medical Education (ACGME) [56]. Given the tendency of prevailing mentoring tools to focus on particular mentoring domains and/or specific phases/stages of the mentoring process and their general failure to consider the inputs of more than one stakeholder’s perspectives, all tools used to assess novice mentoring in undergraduate and postgraduate training in all subspecialties of Internal Medicine were included. Loo et al. [55]’s evidenced based definition of novice mentoring was adopted to focus the search. Only articles published in English, or had English translations, between 1 January 1990 and 31 December 2019 were included.

With guidance from the expert team, the search terms were expanded using Boolean operators to include MeSH and Keywords for all relevant concepts. (S1 Appendix). The broad nature of the research question meant that pilot searches were carried out on variations of the word ‘mentor’ or ‘assessment’ or ‘evaluation’ that appeared in the title or abstract of articles in all accounts of novice mentoring in undergraduate and postgraduate medical training in subspecialties of Internal Medicine as defined by the ACGME [56].

The research team carried out pilot searches of PubMed and OpenGrey databases to determine the appropriateness of the search terms in the pilot searches.

### 3) Select studies to be included in the review

The members of the research team carried out independent searches of Embase, Scopus, ERIC, Cochrane, GreyLit, Web of Science, Open Dissertations and British Education Index databases using similar search strategies. All searches were carried out between 24th April 2018 and 18 October 2018, and 17 December 2019 to 14 February 2020.

**Fig 1:**
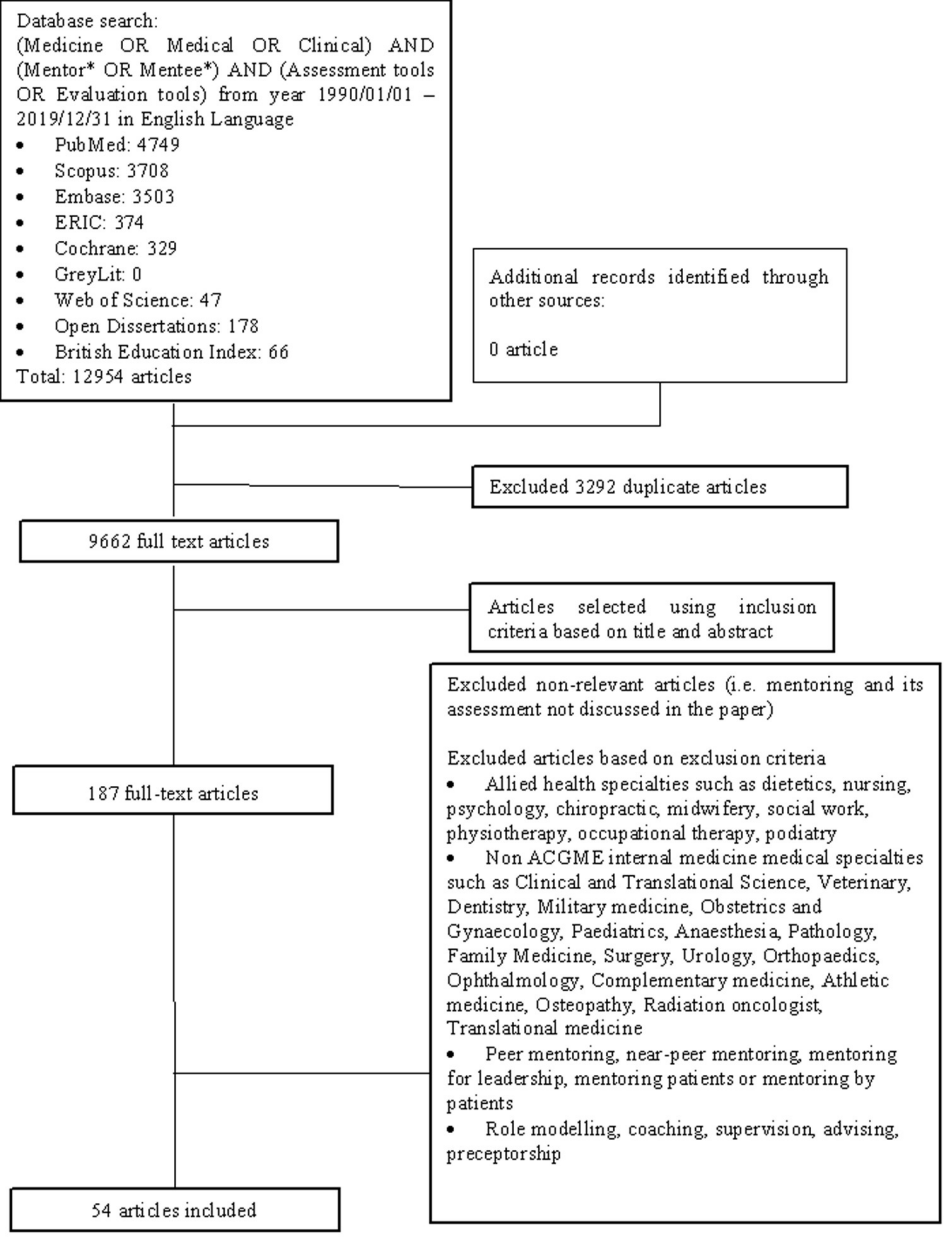
PRISMA flow diagram for search results.

Each member of the research team compiled their own list of articles to be included and compared their results in online discussions with other members of the research team. Sambunjak et al. (2010) [21]’s “negotiated consensual validation” approach to achieve consensus on the final list of articles to be included in the scoping review. A PRISMA diagram was used to represent the search strategy (Fig 1).

### 4) Chart the data

#### Analysis of the manuscripts

To enhance the comprehensiveness of this approach, data from the manuscripts were analyzed using Krishna’s split approach. Krishna’s split approach sees concurrent and independent use of Hsieh and Shannon (2005) approach to directed content analysis approach [57] and Braun and Clarke’s approach to thematic analysis to boost the trustworthiness, reproducibility and accountability of the analysis. Comparisons between the two approaches provides *method triangulation* whilst having each reviewer independently analyse the same data provides *investigator triangulation* [58]. Triangulation also enhances external validity and improves the objectivity within this approach [57]. In addition, consistency between the categories and the themes validate the use of directed content analysis.

These two processes are elaborated in turn below:

*Thematic analysis*. Braun and Clarke [59]’s approach to thematic analysis was adopted to circumnavigate restrictions posed by mentoring’s nature and to scrutinise the characteristics and nature of assessment tools in novice mentoring programs across different clinical, healthcare, educational, healthcare financing and cultural settings, dissimilar mentoring goals and mentee and mentor populations. Braun and Clarke [59]’s approach to thematic analysis also circumnavigates the limitations posed by the wide range of research methodologies present amongst the included articles that prevent the use of statistical pooling and analysis [60, 61]. A thematic analysis is also necessary when studying socio-culturally influenced processes and in the absence of an *a priori* framework of mentoring [51, 62–67].

In phase 1 of Braun and Clarke’s approach, 8 members of the research team (YHW, NYX, ZYKK, KTT, YPT, TXH, SS and LK) carried out independent reviews, ‘actively’ reading the included articles to find meaning and patterns in the data.

In phase 2, ‘codes’ were constructed from the ‘surface’ meaning [4, 59, 68] and collated into a code book to code and analyze the rest of the articles using an iterative step-by-step process. As new codes emerged, these were associated with previous codes and concepts [69].

In phase 3, the categories were organised into themes that best depict the data.

In phase 4, the themes were refined to best represent the whole data set and discussed.

In phase 5, the research team discussed the results of their independent analysis online and at reviewer meetings. “*Negotiated consensual validation*” was used to determine a final list of themes approach [21].

In consultations with the key stakeholders as part of the phase 6 of Levac et al. [48]’s methodological framework, it was suggested that the themes identified were consistent with key elements of mentoring. The expert team also suggested that use of directed content analysis is useful “*when a theory exists about a phenomenon that needs further refinement or development through qualitative research*” [70]. As a result categories were drawn from the most recent reviews of arguably the central aspects of mentoring which were Hee et al. (2019)’s review of mentoring environments [38], Sng et al. (2017) review of mentoring relationships [39] and Tan et al. (2018)’s review of mentoring structures [22] in addition to Krishna et al. (2019)’s account of novice mentoring [54].

*Directed content analysis*. Hsieh and Shannon (2005) approach to directed content analysis approach [57] was employed in three stages [71–73].

Using deductive category application [71, 74], the first stage [71, 72] saw codes drawn from the 4 articles. Drawing upon Mayring (2004) [72]’s account, each code was defined in the code book that contained ‘explicit examples, definitions and rules’ drawn from the data. The code book served to guide the subsequent coding process.

Stage 2 saw the two reviewers using the ‘code book’ to independently extract and code the relevant data from the included articles. Any relevant data not captured by these codes were assigned a new code that was also described in the code book. In keeping with deductive category application [71], coding categories and their definitions were revised. The final codes were compared and discussed with the final author to enhance the reliability of the process [71]. The final author checked the primary data sources to ensure that the codes made sense and were consistently employed. The reviewers and the final author used “*negotiated consensual validation*” to resolve any differences in the coding [21]. The final categories were selected [75] based on whether they appeared in more than 70% of the articles reviewed [76, 77].

Comparisons between the themes identified using Braun and Clarke [59]’s approach and the categories identified from directed content analysis revealed significant consistencies [57].

#### Validity and reliability of the analysis

The split approach adopts an iterative process which meant that any new codes identified was reviewed to verify the classification and ensure complete data extraction. Analysis of all included articles was carried out and discussed online and face-to-face meetings by the independent reviewers. The consensus decisions on the final categories from Hsieh and Shannon (2005) approach to directed content analysis approach [57] and themes from Braun and Clarke [59]’s approach to thematic analysis were reviewed by the last author. The last author also compared the findings of the split approach with prevailing data to ensure theoretical validation.

### 5) Collating, summarising and reporting results

The characteristics of all 54 articles included in this scoping review were tabulated. Details on the author(s), year of publication, study location, Intervention type, and comparator (if any); duration of the intervention, study populations (carer group; care recipient group), aims of the study, methodology, outcome measures and important results were compiled (S2 Appendix). In keeping with Levac et al. [48]’s approach, analysis of the data was focused upon practical areas of interest.

## Results

9,662 unique titles were identified from the nine databases, 187 full-text articles were retrieved, and 54 articles were included in this scoping review. The two categories identified using the split approach include the domains assessed and assessment methods.

### 1. Domains assessed

The 5 domains assessed were the communication, the mentoring process, the mentee’s growth, the mentor and perception of the program.

#### Communication

Mentoring relationships pivot upon effective communications. Nine studies evaluated communications between the mentee and mentor [78–86]. The domains assessed include the type of interactions, be it face-to-face, instant messaging, email, Skype or combinations of these options as well as the frequency and purpose of meetings [78, 80, 83–85, 87, 88].

#### Mentoring process

Five studies evaluated mentee’s and/or mentor’s understanding and expectations of the mentoring process [86, 89–92]. Eight studies assessed the mentee’s mentoring needs to guide recruitment of mentees and mentors decisions and/or guide the mentoring process [83, 86, 92–97]. Five studies evaluated the mentee’s and the mentor’s preferred approach to mentoring to see if there was concordance in their preferences [78, 80, 84, 85, 96]. One study evaluated the impact of the mentoring environment upon a “healthy student faculty interaction” [95]. Six studies assessed the challenges faced during mentoring relationship [82, 86, 88, 97–99].

#### Mentee’s development

15 studies evaluated the mentee’s personal growth by inquiring about their “personal wellbeing”, “development of personality” and “self-perception of their own abilities” [2, 81, 87, 92, 97, 99–108]. 13 studies assessed career development, by evaluating the mentor’s influence as “career guides” and the “impact of faculty relationship on career plans” [2, 8, 36, 79, 92, 99–101, 108–112]. Nine studies assessed the mentee’s clinical performance through appraisal of their acquired skills, improvement in “patient care, medical knowledge and interpersonal skills and communications” and development of academic interests, geared towards specific ACGME competencies [79, 87, 92, 101, 105, 112–115]. Four studies evaluated the mentee’s research development by assessing their research skills and new collaborations [99–101, 116].

#### The mentor

The mentor’s impact upon the mentoring process was evaluated using self-reported changes in practice and the mentee’s perception of the mentor’s abilities. 10 studies [36, 80, 83, 86, 88, 89, 98, 108, 117, 118] assessed the roles of the mentor from the mentee and mentor’s perspective while five studies [36, 81, 88, 91, 119] asked the mentors about their experiences as a mentor.

#### The mentoring program

14 studies assessed the success of the mentoring process by gauging the mentee’s general level of satisfaction with the mentoring program [78, 79, 82–85, 92, 94, 101, 103, 107, 113, 120, 121]. Nine studies [81, 84, 88, 92, 94, 99, 110, 121] assessed mentee satisfaction in the matching process, four studies [84, 91, 94, 122] assessed satisfaction in the host organisation’s funding, incentives and support for mentorship and two studies assessed interests in the program [89, 105] and satisfaction in the length of the mentoring process [78, 80].

### 2. Assessment methods

Assessment methods encapsulated the type of study, the means of collecting data, the mode of measurement, the number of points of evaluation throughout the study, the validity of the tool employed and the target of the tool’s assessment.

S1 Table details the assessment methods of all the included studies (found in the S2 Table). S2 Table summarises the key data collected from the 49 included articles (found in the S2 Table). As shown in S1 and S2 Tables, questionnaires (35%), surveys (26%) and interviews/focus groups (9%) were most commonly used. A mix of the above methods was also common (24%). Additionally, most studies were quantitative (50%), and used Likert scales (37%) or mixed methods (32%). Only a minority of tools were used at more than one time-point (16%) and most of them were unvalidated (78%). It is also of note that the mentee only was the target of such assessment in more than half of all cases (53%) and assessment typically occurs in medical schools (45%) and at university hospitals and/or academic medical centres (49%).

### 6) Undertaking consultations with key stakeholders

Stakeholders were consulted on the findings to garner their inputs on the relative importance and viability of implementing the findings and upon the focus of future studies.

## Discussion

In meetings its objectives, this scoping review highlights the variability in the construct, content, timing, participants involved and focus of prevailing assessment methods in novice mentoring. It also reveals that there is also little by way of consistency in the tools as evidenced by the presence of 49 tools used to assess mentoring. Whilst it is clear that this lack in consistency has a knock-on effect upon practice and oversight of mentoring programs, relationships and processes, in truth it also reflects a more fundamental failure in understanding the mentoring process. In some cases, however, variations in mentoring assessment methods are the result of adaptations to mentoring’s context-dependent and evolving nature whilst others differ by virtue of their settings and goals. These differences often reflect the notion that mentoring is complex and difficult to study holistically or longitudinally predisposing to piecemeal evaluations of ‘areas of interest’ in key areas of mentoring such as communications, the mentee, the mentor, the host and their mentoring relationships. This underpins the presence of makeshift measures in different formats involving specific stakeholders at different times of the mentoring journey.

Reliance upon a constructivist approach and a relativist lens to pull the various socially constructed perspectives together however fails to fully render an effective assessment of the mentoring relationship nor fully capture the changing nature of the mentoring process, the maturing mentoring relationship and the impact of the external influences such as the mentoring environment upon the mentoring environment. This suggests a lack of holistic appraisals of mentoring. It also reaffirms a lack of clarity on the purpose of these assessments.

There is also little by way of explanations of the ‘conceptual foundations’ of the tools [123], compromising understanding of content validity and a dearth of data on the reliability, feasibility and validity data, inter-rater reliability and clinical utility data underline persisting questions as to the validity of what we understand about mentoring as a whole and the theories and program designs built upon prevailing data on the overall data available on mentoring [124, 125].

Indeed, the purpose for the assessments and the role that data from these measures take in influencing the mentoring process also vary. Unsurprisingly, there is little determination as to whether these assessments are formative or summative and few establish the goals of these assessments and how they influence the mentoring process. With little psychometric data, the evidence-based underpinnings and validity of these tools remain questionable particularly given the continued reliance upon self-assessment data at the end of the mentoring process and little by way of frameworks to inculcate formal and informal feedback.

Concurrently, with only 8 out of the 49 tools choosing to evaluate the mentoring process, prevailing data on mentoring progress and the health of mentoring relationships are suspect. This is especially so when the methods used to assess the various domains of the mentoring relationship and program are reliant upon makeshift measures that have not been validated or found to be fit for purpose. Recording of the data collected is also variable and dependent upon whose opinion is sought. There is no consistency in the format of the tool nor little data by way of their validity, feasibility and reliability or their design and theoretical underpinnings.

Furthermore, the majority of studies evaluated mentoring at a single time point, often at the end of the mentoring process where recall bias and the halo effect brought on by a successful mentoring process potentially biases responses.

These findings do little to dissuade concerns that mentoring assessments are flawed, piecemeal and fail to provide an effective picture of the mentee’s, mentor’s and the relationship’s progress and overall condition. These gaps have significant implications upon novice mentoring and mentoring as whole and underline the need for urgent attention.

However, despite prevailing limitations, a preliminary framework may be constructed drawing upon the data accrued thus far. Here, there are guiding ‘considerations’ to consider.

**1. Guiding considerations**
  - The goals of the assessments need to be ascertained
  - Mixed methods approach should be used that captures the participant’s demographics, social, academic, personal, clinical and research backgrounds, experience, goals and motivations. It must also consider the mentoring context and the mentoring goals
  - The assessments should occur throughout the mentoring journey and should include multi-modal assessments, such as surveys, questionnaires, journals and/or interviews, at various junctures of the mentoring process, acknowledging both the various stages of the mentoring process but also the perspectives of ALL the various stakeholders involved
**2. Assessing mentoring dynamics:**
  *a. Mentoring Process:*
    - consider the expectations, motivations and preferred mentoring approach.
    - Assess the knowledge, skills and attitudes of the mentees and the mentors
    - How mentor training and matching of mentors and mentees occurs
    - Determine how mentoring process is initiated and the approach to mentoring.
  *b. Mentoring Relationship:*
    - how mentors and mentees communicate- the form, the frequency, duration and purpose of the meeting.
    - In the face of an evolving mentoring process, there is a need to assess the quality of the mentoring relationships.
**3. Assessing stakeholders:**
  - use *of* multisource feedback collected longitudinally from mentors, mentees and the host organisation to determine how the different stakeholders perform at specific time points and over time.
**4. Assessing outcomes:**
  - This would require longitudinal assessment by the host organisation of the mentoring relationship and mentoring goals
  - Assess satisfaction -feedback in an informal or formal manner, mentor and mentees’ development and growth by self-evaluation and against objective measures.

### Limitations

Without being drawn into prevailing controversies regarding definitions and the role of scoping reviews [46, 50, 125–128], this scoping review’s adoption of Levac, Colquhoun [48]’s pragmatic approach that balances practicality and available resources, did limit the findings of this study [129]. This scoping review is also limited by the presence of a small pool of papers and the preponderance of American and European papers that hinder its generalizability.

However despite these limitations, this scoping review was carried out with the required rigour and transparency advocated by Arksey and O’Malley (2005) [46], Levac et al. [48], Levac et al. [129], Pham et al. (2014) [128] and Tricco et al. (2018) [52] allowing educators and program designers in undergraduate and postgraduate settings a chance to understand the general state of mentoring assessments and we hope help inspire the design of more holistic and longitudinal tools. Perhaps more importantly these findings give weight to the notion that gaps in mentoring assessments are a critical consideration in improving oversight of mentoring processes and preventing mentoring abuse.

## Conclusion

The gaps in the depth and focus of existing tools identified in this scoping review underscore the need for a systematic review to provide a focused and in-depth analysis of mentoring assessment tools [39, 54, 130]. The lessons learnt here lay the foundation for the design of a customised, holistic, longitudinal mentoring assessment tool. In turn, this empowers senior clinicians to provide timely and specific support in relation to the evolving mentoring needs of mentees as they develop their clinical competencies within longitudinal clinical programmes [115, 131].

As such, a modified Delphi is proposed in order to better understand how best to assess the stakeholders (namely, the mentors, mentees and the host organization), the dynamics between them as well as the outcomes of mentoring. This will the next phase of this research project.

## Supporting information

S1 Appendix Search terms. (DOCX)

S2 Appendix Summary of included articles. (DOCX)

S1 Checklist PRISMA 2009 checklist. (PDF)

S1 Table Assessment methods. (DOCX)

S2 Table Key points and summary of domains of mentoring assessed and assessment methods. (DOCX)
