# Supplementary material for: Assessing mentoring: A scoping review of mentoring assessment tools in internal medicine between 1990 and 2019
Source: PLoS One. 2020 May 8;15(5):e0232511. doi: 10.1371/journal.pone.0232511 (PMC7209188; doi:10.1371/journal.pone.0232511)
Supplement: S1 Appendix — (DOCX) [file pone.0232511.s001.docx]

Appendix 1: Search Terms

**Search Terms**

PubMed

(("mentors"[MeSH Terms] OR "mentoring"[MeSH Terms] OR (mentor[Title/Abstract] OR mentor'[Title/Abstract] OR mentor's[Title/Abstract] OR mentor1[Title/Abstract] OR mentor4[Title/Abstract] OR mentorat[Title/Abstract] OR mentore[Title/Abstract] OR mentored[Title/Abstract] OR mentoree[Title/Abstract] OR mentoree's[Title/Abstract] OR mentorees[Title/Abstract] OR mentores[Title/Abstract] OR mentorhood[Title/Abstract] OR mentoria[Title/Abstract] OR mentorial[Title/Abstract] OR mentoring[Title/Abstract] OR mentoring'[Title/Abstract] OR mentoring's[Title/Abstract] OR mentoringaccess[Title/Abstract] OR mentormate[Title/Abstract] OR mentornet[Title/Abstract] OR mentors[Title/Abstract] OR mentors'[Title/Abstract] OR mentorsand[Title/Abstract] OR mentorship[Title/Abstract] OR mentorship'[Title/Abstract] OR mentorships[Title/Abstract] OR mentorsl[Title/Abstract] OR mentortrade[Title/Abstract]) OR mentee[Title/Abstract] OR protege[Title/Abstract] OR "mentor-mentee"[Title/Abstract] OR "mentoring competency"[Title/Abstract]) AND (("Education, Medical, Undergraduate"[MeSH Terms] OR "Students, Medical"[MeSH Terms] OR "education, medical, graduate"[MeSH Terms] OR "Faculty, Medical"[MeSH Terms] OR "Clinical clerkship"[MeSH Terms]) OR ((medical[Title] OR medicine[Title/Abstract] OR clinical[Title/Abstract] OR doctor[Title/Abstract] OR doctors[Title/Abstract]) AND (undergraduate[Title/Abstract] OR student[Title/Abstract] OR students[Title/Abstract] OR trainee[Title/Abstract] OR trainees[Title/Abstract] OR training[Title/Abstract] OR trainer[Title/Abstract] OR trainers[Title/Abstract] OR instruction[Title/Abstract] OR (intern[Title/Abstract] OR intern'[Title/Abstract] OR intern's[Title/Abstract] OR interna[Title/Abstract] OR interna'[Title/Abstract] OR internacao[Title/Abstract] OR internacion[Title/Abstract] OR internacionais[Title/Abstract] OR internacional[Title/Abstract] OR internacionale[Title/Abstract] OR internacionales[Title/Abstract] OR internacionalizacao[Title/Abstract] OR internacionalmente[Title/Abstract] OR internaciones[Title/Abstract] OR internacoes[Title/Abstract] OR internactional[Title/Abstract] OR internada[Title/Abstract] OR internadas[Title/Abstract] OR internadir[Title/Abstract] OR internado[Title/Abstract] OR internados[Title/Abstract] OR internae[Title/Abstract] OR internail[Title/Abstract] OR internaitional[Title/Abstract] OR internal[Title/Abstract] OR internal'[Title/Abstract] OR internal'role[Title/Abstract] OR internal's[Title/Abstract] OR internalbrace[Title/Abstract] OR internalbracetm[Title/Abstract] OR internalbracetrade[Title/Abstract] OR internalcarotid[Title/Abstract] OR internalcolor[Title/Abstract] OR internalcombustion[Title/Abstract] OR internale[Title/Abstract] OR internalexternal[Title/Abstract] OR internalfixation[Title/Abstract] OR internaliation[Title/Abstract] OR internalil[Title/Abstract] OR internalility[Title/Abstract] OR internalimpedance[Title/Abstract] OR internalin[Title/Abstract] OR internalin'[Title/Abstract] OR internalinb[Title/Abstract] OR internalins[Title/Abstract] OR internalional[Title/Abstract] OR internalis[Title/Abstract] OR internalisation[Title/Abstract] OR internalisations[Title/Abstract] OR internalise[Title/Abstract] OR internalised[Title/Abstract] OR internalised'[Title/Abstract] OR internalisers[Title/Abstract] OR internalises[Title/Abstract] OR internalises'[Title/Abstract] OR internalisierende[Title/Abstract] OR internalisierendes[Title/Abstract] OR internalisierung[Title/Abstract] OR internalisierungsprobleme[Title/Abstract] OR internalising[Title/Abstract] OR internalising'[Title/Abstract] OR internalism[Title/Abstract] OR internalist[Title/Abstract] OR internalistic[Title/Abstract] OR internalists[Title/Abstract] OR internalities[Title/Abstract] OR internalitu[Title/Abstract] OR internality[Title/Abstract] OR internality'[Title/Abstract] OR internalizability[Title/Abstract] OR internalizable[Title/Abstract] OR internalizable'[Title/Abstract] OR internalizacion[Title/Abstract] OR internalizada[Title/Abstract] OR internalizado[Title/Abstract] OR internalizador[Title/Abstract] OR internalizados[Title/Abstract] OR internalizaition[Title/Abstract] OR internalizated[Title/Abstract] OR internalizating[Title/Abstract] OR internalization[Title/Abstract] OR internalization'[Title/Abstract] OR internalizations[Title/Abstract] OR internalizaton[Title/Abstract] OR internalize[Title/Abstract] OR internalized[Title/Abstract] OR internalized'[Title/Abstract] OR internalizer[Title/Abstract] OR internalizers[Title/Abstract] OR internalizes[Title/Abstract] OR internalizing[Title/Abstract] OR internalizing'[Title/Abstract] OR internalizing's[Title/Abstract] OR internaliztion[Title/Abstract] OR internaljugular[Title/Abstract] OR internall[Title/Abstract] OR internallcorpus[Title/Abstract] OR internallization[Title/Abstract] OR internally[Title/Abstract] OR internally'[Title/Abstract] OR internalmedical[Title/Abstract] OR internalmedicine[Title/Abstract] OR internalpudendal[Title/Abstract] OR internalradiation[Title/Abstract] OR internalreliability[Title/Abstract] OR internals[Title/Abstract] OR internals'[Title/Abstract] OR internalstructural[Title/Abstract] OR internalstructure[Title/Abstract] OR internaltion[Title/Abstract] OR internaltubing[Title/Abstract] OR internalurethral[Title/Abstract] OR internaly[Title/Abstract] OR internalysis[Title/Abstract] OR internamento[Title/Abstract] OR internamiento[Title/Abstract] OR internamtient[Title/Abstract] OR internanimal[Title/Abstract] OR internanocrystal[Title/Abstract] OR internanodisk[Title/Abstract] OR internanofiber[Title/Abstract] OR internanoparticle[Title/Abstract] OR internanopillar[Title/Abstract] OR internanoplatelet[Title/Abstract] OR internanorod[Title/Abstract] OR internanotube[Title/Abstract] OR internanowell[Title/Abstract] OR internanowire[Title/Abstract] OR internaphthyl[Title/Abstract] OR internaram[Title/Abstract] OR internares[Title/Abstract] OR internarial[Title/Abstract] OR internarional[Title/Abstract] OR internarium[Title/Abstract] OR internas[Title/Abstract] OR internasal[Title/Abstract] OR internasalis[Title/Abstract] OR internasally[Title/Abstract] OR internasals[Title/Abstract] OR internasinus[Title/Abstract] OR internasjonale[Title/Abstract] OR internat[Title/Abstract] OR internat'[Title/Abstract] OR internata[Title/Abstract] OR internatal[Title/Abstract] OR internatally[Title/Abstract] OR internatant[Title/Abstract] OR internate[Title/Abstract] OR internated[Title/Abstract] OR internates[Title/Abstract] OR internatheca[Title/Abstract] OR internati[Title/Abstract] OR internatilization[Title/Abstract] OR internatily[Title/Abstract] OR internatinal[Title/Abstract] OR internatinale[Title/Abstract] OR internatinational[Title/Abstract] OR internatinoal[Title/Abstract] OR internatio[Title/Abstract] OR internatioal[Title/Abstract] OR internatioanl[Title/Abstract] OR internatiol[Title/Abstract] OR internation[Title/Abstract] OR internation's[Title/Abstract] OR internationa[Title/Abstract] OR internationaal[Title/Abstract] OR internationai[Title/Abstract] OR international[Title/Abstract] OR international'[Title/Abstract] OR international's[Title/Abstract] OR internationala[Title/Abstract] OR internationalagency[Title/Abstract] OR internationalclassification[Title/Abstract] OR internationalcouncil[Title/Abstract] OR internationaldayofradiology[Title/Abstract] OR internationaldentalcharts[Title/Abstract] OR internationaldiabetes[Title/Abstract] OR internationaldrugsurvey[Title/Abstract] OR internationale[Title/Abstract] OR internationale's[Title/Abstract] OR internationaled[Title/Abstract] OR internationaled'[Title/Abstract] OR internationalefms[Title/Abstract] OR internationalen[Title/Abstract] OR internationaler[Title/Abstract] OR internationales[Title/Abstract] OR internationaleye[Title/Abstract] OR internationalgenome[Title/Abstract] OR internationalhealthpartnership[Title/Abstract] OR internationalindex[Title/Abstract] OR internationalis[Title/Abstract] OR internationalisation[Title/Abstract] OR internationalisation'[Title/Abstract] OR internationalise[Title/Abstract] OR internationalised[Title/Abstract] OR internationalising[Title/Abstract] OR internationalism[Title/Abstract] OR internationalisme[Title/Abstract] OR internationalist[Title/Abstract] OR internationalistic[Title/Abstract] OR internationalists[Title/Abstract] OR internationalists'[Title/Abstract] OR internationality[Title/Abstract] OR internationalizable[Title/Abstract] OR internationalizaition[Title/Abstract] OR internationalization[Title/Abstract] OR internationalization'[Title/Abstract] OR internationalize[Title/Abstract] OR internationalized[Title/Abstract] OR internationalized'[Title/Abstract] OR internationalizes[Title/Abstract] OR internationalizing[Title/Abstract] OR internationall[Title/Abstract] OR internationally[Title/Abstract] OR internationallyagreed[Title/Abstract] OR internationallyrecognized[Title/Abstract] OR internationalmodified[Title/Abstract] OR internationalmulticenter[Title/Abstract] OR internationalorganization[Title/Abstract] OR internationalorganizations[Title/Abstract] OR internationalregistry[Title/Abstract] OR internationalresearch[Title/Abstract] OR internationals[Title/Abstract] OR internationalschoolhealt[Title/Abstract] OR internationalsociety[Title/Abstract] OR internationalsymposium[Title/Abstract] OR internationaly[Title/Abstract] OR internationational[Title/Abstract] OR internationaux[Title/Abstract] OR internationbal[Title/Abstract] OR internationellt[Title/Abstract] OR internationial[Title/Abstract] OR internationist[Title/Abstract] OR internationization[Title/Abstract] OR internationl[Title/Abstract] OR internationlale[Title/Abstract] OR internationnal[Title/Abstract] OR internationnally[Title/Abstract] OR internationnaly[Title/Abstract] OR internations[Title/Abstract] OR internationsl[Title/Abstract] OR internatioval[Title/Abstract] OR internatization[Title/Abstract] OR internatl[Title/Abstract] OR internatlization[Title/Abstract] OR internato[Title/Abstract] OR internatoional[Title/Abstract] OR internatonal[Title/Abstract] OR internatsional'naia[Title/Abstract] OR internatsional'nyl[Title/Abstract] OR internattional[Title/Abstract] OR internatuionalis[Title/Abstract] OR internatus[Title/Abstract] OR internauts[Title/Abstract] OR internavec[Title/Abstract] OR internazionale[Title/Abstract] OR internazionali[Title/Abstract] OR interne[Title/Abstract] OR interne'[Title/Abstract] OR interne's[Title/Abstract] OR internebulizer[Title/Abstract] OR internecine[Title/Abstract] OR internecrosectomy[Title/Abstract] OR interned[Title/Abstract] OR internediate[Title/Abstract] OR internee[Title/Abstract] OR interneedle[Title/Abstract] OR internees[Title/Abstract] OR internees'[Title/Abstract] OR internegative[Title/Abstract] OR internegatives[Title/Abstract] OR interneighbor[Title/Abstract] OR interneighborhood[Title/Abstract] OR internelized[Title/Abstract] OR internement[Title/Abstract] OR internen[Title/Abstract] OR internention[Title/Abstract] OR interneocortical[Title/Abstract] OR interneonatal[Title/Abstract] OR interneonate[Title/Abstract] OR interneoplastic[Title/Abstract] OR internephro[Title/Abstract] OR internephron[Title/Abstract] OR internephronal[Title/Abstract] OR interner[Title/Abstract] OR internerneurons[Title/Abstract] OR internerons[Title/Abstract] OR interneruon[Title/Abstract] OR internerval[Title/Abstract] OR internerve[Title/Abstract] OR internervous[Title/Abstract] OR internes[Title/Abstract] OR interneship[Title/Abstract] OR interneships[Title/Abstract] OR internest[Title/Abstract] OR internested[Title/Abstract] OR internesting[Title/Abstract] OR internet[Title/Abstract] OR internet'[Title/Abstract] OR internet's[Title/Abstract] OR internet2[Title/Abstract] OR internetadvance[Title/Abstract] OR internetbased[Title/Abstract] OR internetbasiert[Title/Abstract] OR internetbasierte[Title/Abstract] OR internetbeteg[Title/Abstract] OR internetdatabase[Title/Abstract] OR internete[Title/Abstract] OR interneterziehung[Title/Abstract] OR internetfor[Title/Abstract] OR internetgebrauch[Title/Abstract] OR internetinternet[Title/Abstract] OR internetional[Title/Abstract] OR internetis[Title/Abstract] OR internetization[Title/Abstract] OR internetnutzung[Title/Abstract] OR internetnutzungskompetenz[Title/Abstract] OR internetowej[Title/Abstract] OR internetowych[Title/Abstract] OR internetpresentations[Title/Abstract] OR internetquestion[Title/Abstract] OR internets[Title/Abstract] OR internets'[Title/Abstract] OR internetserver[Title/Abstract] OR internetservices[Title/Abstract] OR internetsex[Title/Abstract] OR internetspielstorung[Title/Abstract] OR internetsucht[Title/Abstract] OR internettreatment[Title/Abstract] OR internetu[Title/Abstract] OR internetwork[Title/Abstract] OR internetworked[Title/Abstract] OR internetworking[Title/Abstract] OR internetworks[Title/Abstract] OR internetworldstats[Title/Abstract] OR interneu[Title/Abstract] OR interneulrons[Title/Abstract] OR interneuones[Title/Abstract] OR interneuons[Title/Abstract] OR interneural[Title/Abstract] OR interneurial[Title/Abstract] OR interneurite[Title/Abstract] OR interneuritic[Title/Abstract] OR interneuroblastic[Title/Abstract] OR interneurocompartmental[Title/Abstract] OR interneurofilament[Title/Abstract] OR interneurogenesis[Title/Abstract] OR interneuromast[Title/Abstract] OR interneuromastic[Title/Abstract] OR interneuromeric[Title/Abstract] OR interneuron[Title/Abstract] OR interneuron's[Title/Abstract] OR interneuronal[Title/Abstract] OR interneuronally[Title/Abstract] OR interneurone[Title/Abstract] OR interneurone'[Title/Abstract] OR interneuroneal[Title/Abstract] OR interneurones[Title/Abstract] OR interneurones'[Title/Abstract] OR interneuronic[Title/Abstract] OR interneuronitis[Title/Abstract] OR interneuronogenesis[Title/Abstract] OR interneuronopathies[Title/Abstract] OR interneuronopathies'[Title/Abstract] OR interneuronopathy[Title/Abstract] OR interneuronopathy'[Title/Abstract] OR interneurons[Title/Abstract] OR interneurons'[Title/Abstract] OR interneurons'at[Title/Abstract] OR interneuronsin[Title/Abstract] OR interneurosn[Title/Abstract] OR interneutros[Title/Abstract] OR internexal[Title/Abstract] OR internexin[Title/Abstract] OR internexin's[Title/Abstract] OR interngal[Title/Abstract] OR interni[Title/Abstract] OR interniche[Title/Abstract] OR internicola[Title/Abstract] OR internight[Title/Abstract] OR internii[Title/Abstract] OR interniloculus[Title/Abstract] OR interning[Title/Abstract] OR internipple[Title/Abstract] OR internis[Title/Abstract] OR internische[Title/Abstract] OR interniship[Title/Abstract] OR internist[Title/Abstract] OR internist'[Title/Abstract] OR internist's[Title/Abstract] OR internista[Title/Abstract] OR internistas[Title/Abstract] OR interniste[Title/Abstract] OR internisten[Title/Abstract] OR internistendagen[Title/Abstract] OR internistenkongress[Title/Abstract] OR internistenwoche[Title/Abstract] OR internisti[Title/Abstract] OR internistic[Title/Abstract] OR internistical[Title/Abstract] OR internistically[Title/Abstract] OR internistinnen[Title/Abstract] OR internistis[Title/Abstract] OR internistische[Title/Abstract] OR internistischer[Title/Abstract] OR internists[Title/Abstract] OR internists'[Title/Abstract] OR internitrogen[Title/Abstract] OR internitroxide[Title/Abstract] OR internittens[Title/Abstract] OR internittent[Title/Abstract] OR internity[Title/Abstract] OR internization[Title/Abstract] OR internizes[Title/Abstract] OR internl[Title/Abstract] OR internleukin[Title/Abstract] OR internlingua[Title/Abstract] OR internlized[Title/Abstract] OR internmediate[Title/Abstract] OR internmedicin[Title/Abstract] OR internment[Title/Abstract] OR internment'[Title/Abstract] OR internments[Title/Abstract] OR internmucleosomal[Title/Abstract] OR internnected[Title/Abstract] OR internnn[Title/Abstract] OR internnual[Title/Abstract] OR interno[Title/Abstract] OR interno'[Title/Abstract] OR internodal[Title/Abstract] OR internodalfocal[Title/Abstract] OR internodally[Title/Abstract] OR internodals[Title/Abstract] OR internode[Title/Abstract] OR internode's[Title/Abstract] OR internode1[Title/Abstract] OR internode2[Title/Abstract] OR internodel[Title/Abstract] OR internodense[Title/Abstract] OR internodes[Title/Abstract] OR internodes'[Title/Abstract] OR internodia[Title/Abstract] OR internodial[Title/Abstract] OR internodiales[Title/Abstract] OR internodii[Title/Abstract] OR internodium[Title/Abstract] OR internodons[Title/Abstract] OR internodopathy[Title/Abstract] OR internodular[Title/Abstract] OR internodule[Title/Abstract] OR internodules[Title/Abstract] OR internoes[Title/Abstract] OR internogastricus[Title/Abstract] OR internoise[Title/Abstract] OR internolaterally[Title/Abstract] OR internology[Title/Abstract] OR internolol[Title/Abstract] OR internomological[Title/Abstract] OR internopyramidal[Title/Abstract] OR internordic[Title/Abstract] OR internoretinal[Title/Abstract] OR internoretinalis[Title/Abstract] OR internormalized[Title/Abstract] OR internorum[Title/Abstract] OR internos[Title/Abstract] OR internosological[Title/Abstract] OR internostril[Title/Abstract] OR internotch[Title/Abstract] OR internote[Title/Abstract] OR internoteniate[Title/Abstract] OR internous[Title/Abstract] OR internparent[Title/Abstract] OR interns[Title/Abstract] OR interns'[Title/Abstract] OR interns'ability[Title/Abstract] OR internship[Title/Abstract] OR internship'[Title/Abstract] OR internship's[Title/Abstract] OR internships[Title/Abstract] OR internships'[Title/Abstract] OR internsifier[Title/Abstract] OR internsity[Title/Abstract] OR interntal[Title/Abstract] OR interntational[Title/Abstract] OR interntestinal[Title/Abstract] OR interntion[Title/Abstract] OR interntional[Title/Abstract] OR internucial[Title/Abstract] OR internuclar[Title/Abstract] OR internuclear[Title/Abstract] OR internuclearis[Title/Abstract] OR internuclearly[Title/Abstract] OR internuclei[Title/Abstract] OR internucleobase[Title/Abstract] OR internucleoid[Title/Abstract] OR internucleolar[Title/Abstract] OR internucleon[Title/Abstract] OR internucleoside[Title/Abstract] OR internucleosidic[Title/Abstract] OR internucleosoma[Title/Abstract] OR internucleosomal[Title/Abstract] OR internucleosomally[Title/Abstract] OR internucleosomalsize[Title/Abstract] OR internucleosome[Title/Abstract] OR internucleosomic[Title/Abstract] OR internucleotic[Title/Abstract] OR internucleotide[Title/Abstract] OR internucleotidec[Title/Abstract] OR internucleotidic[Title/Abstract] OR internucleotidyl[Title/Abstract] OR internuclesomal[Title/Abstract] OR internuclesosomal[Title/Abstract] OR internucleus[Title/Abstract] OR internuerons[Title/Abstract] OR internuittent[Title/Abstract] OR internulceosomal[Title/Abstract] OR internull[Title/Abstract] OR internullo[Title/Abstract] OR internum[Title/Abstract] OR internuncal[Title/Abstract] OR internuncial[Title/Abstract] OR internuncials[Title/Abstract] OR internurse[Title/Abstract] OR internursing[Title/Abstract] OR internus[Title/Abstract] OR internut[Title/Abstract] OR internvals[Title/Abstract] OR internventions[Title/Abstract] OR internvetions[Title/Abstract] OR internvetions'[Title/Abstract] OR internwebben[Title/Abstract]) OR clerkship[Title] OR (residen[Title/Abstract] OR residence[Title/Abstract] OR residence'[Title/Abstract] OR residence's[Title/Abstract] OR residenced[Title/Abstract] OR residencedagger[Title/Abstract] OR residencein[Title/Abstract] OR residences[Title/Abstract] OR residences'[Title/Abstract] OR residencia[Title/Abstract] OR residencial[Title/Abstract] OR residenciales[Title/Abstract] OR residencias[Title/Abstract] OR residencies[Title/Abstract] OR residencies'[Title/Abstract] OR residencing[Title/Abstract] OR residencuy[Title/Abstract] OR residency[Title/Abstract] OR residency'[Title/Abstract] OR residency's[Title/Abstract] OR residencyprograms[Title/Abstract] OR residencyresults[Title/Abstract] OR residencyteam[Title/Abstract] OR residencytraining[Title/Abstract] OR residene[Title/Abstract] OR residens's[Title/Abstract] OR residense[Title/Abstract] OR resident[Title/Abstract] OR resident'[Title/Abstract] OR resident's[Title/Abstract] OR resident`s[Title/Abstract] OR residentail[Title/Abstract] OR residental[Title/Abstract] OR residentas[Title/Abstract] OR residente[Title/Abstract] OR residented[Title/Abstract] OR residentes[Title/Abstract] OR residentes'[Title/Abstract] OR residentesaeu[Title/Abstract] OR residentfirst[Title/Abstract] OR residenti[Title/Abstract] OR residentia[Title/Abstract] OR residential[Title/Abstract] OR residential'[Title/Abstract] OR residentialagedcaresector[Title/Abstract] OR residentiality[Title/Abstract] OR residentially[Title/Abstract] OR residentials[Title/Abstract] OR residentiary[Title/Abstract] OR residentiel[Title/Abstract] OR residentielles[Title/Abstract] OR residentiels[Title/Abstract] OR residenting[Title/Abstract] OR residentphysicians[Title/Abstract] OR residentproteins[Title/Abstract] OR residentratiostaff[Title/Abstract] OR residents[Title/Abstract] OR residents'[Title/Abstract] OR residents'awareness[Title/Abstract] OR residents'characteristics[Title/Abstract] OR residents'corner[Title/Abstract] OR residents'depression[Title/Abstract] OR residents'disease[Title/Abstract] OR residents'health[Title/Abstract] OR residents'infection[Title/Abstract] OR residents'new[Title/Abstract] OR residents'own[Title/Abstract] OR residents'physical[Title/Abstract] OR residents'room[Title/Abstract] OR residents's[Title/Abstract] OR residents'strike[Title/Abstract] OR residents39[Title/Abstract] OR residents`expressions[Title/Abstract] OR residents`knowledge[Title/Abstract] OR residents`motivation[Title/Abstract] OR residentsare[Title/Abstract] OR residentsgave[Title/Abstract] OR residentship[Title/Abstract] OR residentsresponsible[Title/Abstract] OR residentstaff[Title/Abstract] OR residentswas[Title/Abstract] OR residentswith[Title/Abstract] OR residentteachers[Title/Abstract] OR residenvial[Title/Abstract] OR residenza[Title/Abstract] OR residenze[Title/Abstract] OR residenze'[Title/Abstract] OR residenzialita[Title/Abstract]) OR practice[Title/Abstract] OR learning[Title/Abstract] OR (competen[Title/Abstract] OR competenc[Title/Abstract] OR competence[Title/Abstract] OR competence'[Title/Abstract] OR competence''[Title/Abstract] OR competence's[Title/Abstract] OR competencecompared[Title/Abstract] OR competencee[Title/Abstract] OR competencein[Title/Abstract] OR competences[Title/Abstract] OR competences'[Title/Abstract] OR competenceto[Title/Abstract] OR competencewhen[Title/Abstract] OR competencia[Title/Abstract] OR competencial[Title/Abstract] OR competencias[Title/Abstract] OR competencie[Title/Abstract] OR competencies[Title/Abstract] OR competencies'[Title/Abstract] OR competencies''[Title/Abstract] OR competency[Title/Abstract] OR competency'[Title/Abstract] OR competency's[Title/Abstract] OR competencyas[Title/Abstract] OR competencybased[Title/Abstract] OR competendes[Title/Abstract] OR competene[Title/Abstract] OR competent[Title/Abstract] OR competent'[Title/Abstract] OR competentce[Title/Abstract] OR competente[Title/Abstract] OR competentes[Title/Abstract] OR competenti[Title/Abstract] OR competentie[Title/Abstract] OR competention[Title/Abstract] OR competently[Title/Abstract] OR competents[Title/Abstract] OR competenze[Title/Abstract] OR competenzzentrum[Title/Abstract]) OR programme[Title/Abstract] OR programmes[Title/Abstract] OR program[Title/Abstract] OR programs[Title/Abstract] OR teaching[Title/Abstract] OR education[Title/Abstract] OR graduate[Title/Abstract] OR graduates[Title/Abstract] OR internship[Title/Abstract] OR residency[Title/Abstract] OR "internship and residency"[MeSH Terms] OR academic[Title/Abstract])))) AND ("Educational Measurement"[Mesh] OR "Program Evaluation"[Mesh] OR (focus group[tiab] OR focus groups[tiab]) OR (survey[tiab] OR survey'[tiab] OR survey's[tiab] OR survey100[tiab] OR survey12[tiab] OR survey123[tiab] OR survey1988[tiab] OR survey2010[tiab] OR survey226[tiab] OR survey36[tiab] OR surveyability[tiab] OR surveyable[tiab] OR surveyance[tiab] OR surveyant's[tiab] OR surveybetween[tiab] OR surveyd[tiab] OR surveydagger[tiab] OR surveydata[tiab] OR surveydelhi[tiab] OR surveydesigncross[tiab] OR surveyed[tiab] OR surveyedandtestedthe[tiab] OR surveyedpopulation[tiab] OR surveyees[tiab] OR surveyelicited[tiab] OR surveyer[tiab] OR surveyes[tiab] OR surveyeyed[tiab] OR surveyform[tiab] OR surveyfreq[tiab] OR surveygizmo[tiab] OR surveyin[tiab] OR surveying[tiab] OR surveying'[tiab] OR surveyings[tiab] OR surveylogistic[tiab] OR surveymaster[tiab] OR surveymeans[tiab] OR surveymeter[tiab] OR surveymonkey[tiab] OR surveymonkey's[tiab] OR surveymonkeytrade[tiab] OR surveyng[tiab] OR surveyor[tiab] OR surveyor'[tiab] OR surveyor's[tiab] OR surveyors[tiab] OR surveyors'[tiab] OR surveyortrade[tiab] OR surveyparticipantsresidents[tiab] OR surveyphreg[tiab] OR surveyplus[tiab] OR surveyprocess[tiab] OR surveyreg[tiab] OR surveyrobotic[tiab] OR surveys[tiab] OR surveys'[tiab] OR surveys'food[tiab] OR surveys'usefulness[tiab] OR surveysclub[tiab] OR surveyselect[tiab] OR surveyset[tiab] OR surveyset'[tiab] OR surveyspot[tiab] OR surveystrade[tiab] OR surveysuite[tiab] OR surveytaken[tiab] OR surveythese[tiab] OR surveytm[tiab] OR surveytracker[tiab] OR surveytrade[tiab] OR surveyvas[tiab] OR surveywas[tiab] OR surveywiz[tiab] OR surveyxact[tiab]) OR (questionnaires[tiab] OR questionnaires'[tiab] OR questionnaires''[tiab] OR questionnairescan[tiab] OR questionnaireshort[tiab] OR questionnairess[tiab]) OR (feedback[tiab] OR feedback'[tiab] OR feedback''[tiab] OR feedback's[tiab] OR feedbackdesign[tiab] OR feedbacked[tiab] OR feedbackfor[tiab] OR feedbackin[tiab] OR feedbacking[tiab] OR feedbackly[tiab] OR feedbackon[tiab] OR feedbackrehabilitation[tiab] OR feedbacks[tiab] OR feedbacks'[tiab] OR feedbacksystem[tiab] OR feedbackward[tiab]) OR (interview[tiab] OR interview'[tiab] OR interview's[tiab] OR interview'with[tiab] OR interview194[tiab] OR interviewability[tiab] OR interviewable[tiab] OR interviewable'[tiab] OR interviewadministered[tiab] OR interviewbased[tiab] OR interviewbroker[tiab] OR interviewd[tiab] OR interviewdata[tiab] OR interviewe[tiab] OR interviewed[tiab] OR interviewed'[tiab] OR interviewedduring[tiab] OR interviewedfor[tiab] OR intervieweds[tiab] OR interviewee[tiab] OR interviewee's[tiab] OR interviewee'speeches[tiab] OR interviewees[tiab] OR interviewees'[tiab] OR interviewer[tiab] OR interviewer'[tiab] OR interviewer's[tiab] OR interviewer3[tiab] OR intervieweradministered[tiab] OR interviewerd[tiab] OR interviewers[tiab] OR interviewers'[tiab] OR interviewes[tiab] OR interviewguide[tiab] OR interviewin[tiab] OR interviewing[tiab] OR interviewing'[tiab] OR interviewing's[tiab] OR interviewingfor[tiab] OR interviewleitfaden[tiab] OR interviewpatient[tiab] OR interviews[tiab] OR interviews'[tiab] OR interviews1[tiab] OR interviews3d[tiab] OR interviewsfrom[tiab] OR interviewshowed[tiab] OR interviewsurvey[tiab] OR interviewsusing[tiab] OR interviewtext[tiab] OR interviewwas[tiab] OR interviewwd[tiab]) OR (assessment[tiab] OR assessment'[tiab] OR assessment''[tiab] OR assessment's[tiab] OR assessment2[tiab] OR assessment75[tiab] OR assessment8[tiab] OR assessmenta[tiab] OR assessmentand[tiab] OR assessmentandfitness[tiab] OR assessmentbased[tiab] OR assessmentbeta[tiab] OR assessmentboards[tiab] OR assessmentcenter[tiab] OR assessmentchanges[tiab] OR assessmentclinical[tiab] OR assessmentdagger[tiab] OR assessmentdepends[tiab] OR assessmentdescriptors[tiab] OR assessmentestimated[tiab] OR assessmentfactor[tiab] OR assessmentfor[tiab] OR assessmentgpa[tiab] OR assessmentgsc[tiab] OR assessmentidi[tiab] OR assessmentin[tiab] OR assessmenting[tiab] OR assessmentinsulin[tiab] OR assessmentmanagement[tiab] OR assessmento[tiab] OR assessmentof[tiab] OR assessmentofcurrent[tiab] OR assessmentphysical[tiab] OR assessmentpop[tiab] OR assessmentprocess[tiab] OR assessmentresult[tiab] OR assessmentrevealed[tiab] OR assessments[tiab] OR assessments'[tiab] OR assessments1[tiab] OR assessmentshavenot[tiab] OR assessmentsm[tiab] OR assessmentsummary[tiab] OR assessmentsurgical[tiab] OR assessmentteam[tiab] OR assessmentthe[tiab] OR assessmenttherapy[tiab] OR assessmentthere[tiab] OR assessmentthis[tiab] OR assessmenttls[tiab] OR assessmenttm[tiab] OR assessmenttool[tiab] OR assessmenttools[tiab] OR assessmenttrade[tiab] OR assessmentunder[tiab] OR assessmentunits[tiab] OR assessmentwencke[tiab] OR assessmentwhile[tiab]) OR (evaluation[tiab] OR evaluation'[tiab] OR evaluation''[tiab] OR evaluation's[tiab] OR evaluation1[tiab] OR evaluational[tiab] OR evaluationand[tiab] OR evaluationappropriate[tiab] OR evaluationary[tiab] OR evaluationdagger[tiab] OR evaluatione[tiab] OR evaluationed[tiab] OR evaluationelective[tiab] OR evaluationfollowing[tiab] OR evaluationfor[tiab] OR evaluationg[tiab] OR evaluationgrade[tiab] OR evaluationicuintensive[tiab] OR evaluationii[tiab] OR evaluationiii[tiab] OR evaluationin[tiab] OR evaluationism[tiab] OR evaluationism'[tiab] OR evaluationl[tiab] OR evaluationof[tiab] OR evaluationon[tiab] OR evaluationproblem[tiab] OR evaluationrevealed[tiab] OR evaluations[tiab] OR evaluations'[tiab] OR evaluationsfragebogen[tiab] OR evaluationsincluded[tiab] OR evaluationsinstrument[tiab] OR evaluationsmethoden[tiab] OR evaluationsrevealed[tiab] OR evaluationss[tiab] OR evaluationsstudie[tiab] OR evaluationssysteme[tiab] OR evaluationsystem[tiab] OR evaluationsystems[tiab] OR evaluationt[tiab] OR evaluationtions[tiab] OR evaluationto[tiab] OR evaluationtool[tiab] OR evaluationwas[tiab]) OR (measurement[tiab] OR measurement'[tiab] OR measurement's[tiab] OR measurement1[tiab] OR measuremental[tiab] OR measurementd[tiab] OR measuremented[tiab] OR measurementexhaled[tiab] OR measurementf[tiab] OR measurementin[tiab] OR measurementinstrumentation[tiab] OR measuremention[tiab] OR measurementis[tiab] OR measurementkomputation[tiab] OR measurementl[tiab] OR measurementmanometry[tiab] OR measurementmaterial[tiab] OR measurementmethods[tiab] OR measurementof[tiab] OR measurementon[tiab] OR measurementorfact[tiab] OR measurementpro[tiab] OR measurementresults[tiab] OR measurements[tiab] OR measurements'[tiab] OR measurements's[tiab] OR measurements0[tiab] OR measurements5[tiab] OR measurementsa[tiab] OR measurementsare[tiab] OR measurementsbetween[tiab] OR measurementscanbe[tiab] OR measurementscheme[tiab] OR measurementsfor[tiab] OR measurementsgave[tiab] OR measurementsin[tiab] OR measurementsindicate[tiab] OR measurementsments[tiab] OR measurementsmoking[tiab] OR measurementsof[tiab] OR measurementson[tiab] OR measurementsreveal[tiab] OR measurementss[tiab] OR measurementsv3[tiab] OR measurementswere[tiab] OR measurementsystem[tiab] OR measurementtime[tiab] OR measurementtrade[tiab] OR measurementts[tiab] OR measurementusing[tiab] OR measurementwas[tiab] OR measurementws[tiab])) AND (("1990/01/01"[PDAT] : "2019/12/31"[PDAT]) AND English[lang])

Scopus

( TITLE-ABS ( mentor* OR mentee OR protege OR mentor-mentee OR "mentoring competency" ) ) AND ( ( TITLE-ABS ( medical OR medicine OR clinical OR doctor OR doctors ) ) AND ( TITLE-ABS ( undergraduate OR student OR trainee OR training OR trainer OR instruction OR intern* OR clerkship OR residen* OR practice OR learning OR competen* OR programme OR program OR teaching OR education OR learning OR graduate OR student OR students OR internship OR residency OR clerkship OR academic ) ) ) AND ( ( ( ( TITLE-ABS ( assessment* OR evaluation* OR "Evaluation Measurement" OR "Program Evaluation" OR "Focus group" OR survey* OR feedback* OR interview* OR measurement* ) ) ) ) ) AND ( LIMIT-TO ( PUBYEAR , 2019 ) OR LIMIT-TO ( PUBYEAR , 2018 ) OR LIMIT-TO ( PUBYEAR , 2017 ) OR LIMIT-TO ( PUBYEAR , 2016 ) OR LIMIT-TO ( PUBYEAR , 2015 ) OR LIMIT-TO ( PUBYEAR , 2014 ) OR LIMIT-TO ( PUBYEAR , 2013 ) OR LIMIT-TO ( PUBYEAR , 2012 ) OR LIMIT-TO ( PUBYEAR , 2011 ) OR LIMIT-TO ( PUBYEAR , 2010 ) OR LIMIT-TO ( PUBYEAR , 2009 ) OR LIMIT-TO ( PUBYEAR , 2008 ) OR LIMIT-TO ( PUBYEAR , 2007 ) OR LIMIT-TO ( PUBYEAR , 2006 ) OR LIMIT-TO ( PUBYEAR , 2005 ) OR LIMIT-TO ( PUBYEAR , 2004 ) OR LIMIT-TO ( PUBYEAR , 2003 ) OR LIMIT-TO ( PUBYEAR , 2002 ) OR LIMIT-TO ( PUBYEAR , 2001 ) OR LIMIT-TO ( PUBYEAR , 2000 ) OR LIMIT-TO ( PUBYEAR , 1999 ) OR LIMIT-TO ( PUBYEAR , 1998 ) OR LIMIT-TO ( PUBYEAR , 1997 ) OR LIMIT-TO ( PUBYEAR , 1996 ) OR LIMIT-TO ( PUBYEAR , 1995 ) OR LIMIT-TO ( PUBYEAR , 1994 ) OR LIMIT-TO ( PUBYEAR , 1993 ) OR LIMIT-TO ( PUBYEAR , 1992 ) OR LIMIT-TO ( PUBYEAR , 1991 ) OR LIMIT-TO ( PUBYEAR , 1990 ) ) AND ( LIMIT-TO ( LANGUAGE , "English" )4

ERIC

(SU.EXACT.EXPLODE("Mentors") OR ti,ab(mentor* OR mentee OR protege OR mentor-mentee OR "mentoring competency")) AND ((SU.EXACT("Medical Education") OR SU.EXACT("Graduate Medical Education")) OR (ti,ab(medical OR medicine OR clinical OR doctor OR doctors) AND ti,ab(undergraduate OR student OR trainee OR training OR trainer OR instruction OR intern* OR clerkship OR residen* OR practice OR learning OR competen* OR programme OR program OR teaching OR education OR learning OR graduate OR student OR students OR internship OR residency OR clerkship OR academic))) AND ((SU.EXACT.EXPLODE("Educational Indicators") OR SU.EXACT.EXPLODE(“Program Evaluation”)) OR ti,ab(“Focus group” OR Survey* OR Questionnaires* OR feedback* OR interview* OR Assessment* OR Evaluation* OR Measurement*))

EMBASE

('mentoring'/exp OR 'mentoring':ti,ab OR mentor*:ti,ab OR mentee:ti,ab OR mentees:ti,ab OR protege:ti,ab OR 'mentor mentee':ti,ab OR 'mentoring competency':ti,ab) AND (('medical education'/exp OR 'medical education':ti,ab OR 'medical school'/exp OR 'medical school':ti,ab OR 'clinical education'/exp OR 'clinical education':ti,ab) AND [embase]/lim OR ((medical:ti,ab OR medicine:ti,ab OR doctor:ti,ab OR doctors:ti,ab OR clinical:ti,ab) AND (undergraduate:ti,ab OR student:ti,ab OR trainee:ti,ab OR trainess:ti,ab OR training:ti,ab OR trainer:ti,ab OR trainers:ti,ab OR instruction:ti,ab OR intern*:ti,ab OR clerkship:ti,ab OR residen*:ti,ab OR practice:ti,ab OR competen*:ti,ab OR programme:ti,ab OR programmes:ti,ab OR program:ti,ab OR programs:ti,ab OR teaching:ti,ab OR learning:ti,ab OR academic:ti,ab OR education:ti,ab OR internship:ti,ab OR residency:ti,ab) AND [embase]/lim)) AND [embase]/lim NOT [medline]/lim AND (('educational measurement'/exp OR 'program evaluation'/exp) OR ('focus group' OR survey* OR questionnaires* OR feedback* OR interview* OR assessment* OR evaluation* OR measurement*): ti,ab) AND (1990:py OR 1991:py OR 1992:py OR 1993:py OR 1994:py OR 1995:py OR 1996:py OR 1997:py OR 1998:py OR 1999:py OR 2000:py OR 2001:py OR 2002:py OR 2003:py OR 2004:py OR 2005:py OR 2006:py OR 2007:py OR 2008:py OR 2009:py OR 2010:py OR 2011:py OR 2012:py OR 2013:py OR 2014:py OR 2015:py OR 2016:py OR 2017:py OR 2018:py OR 2019:py) AND [english]/lim

Cochrane


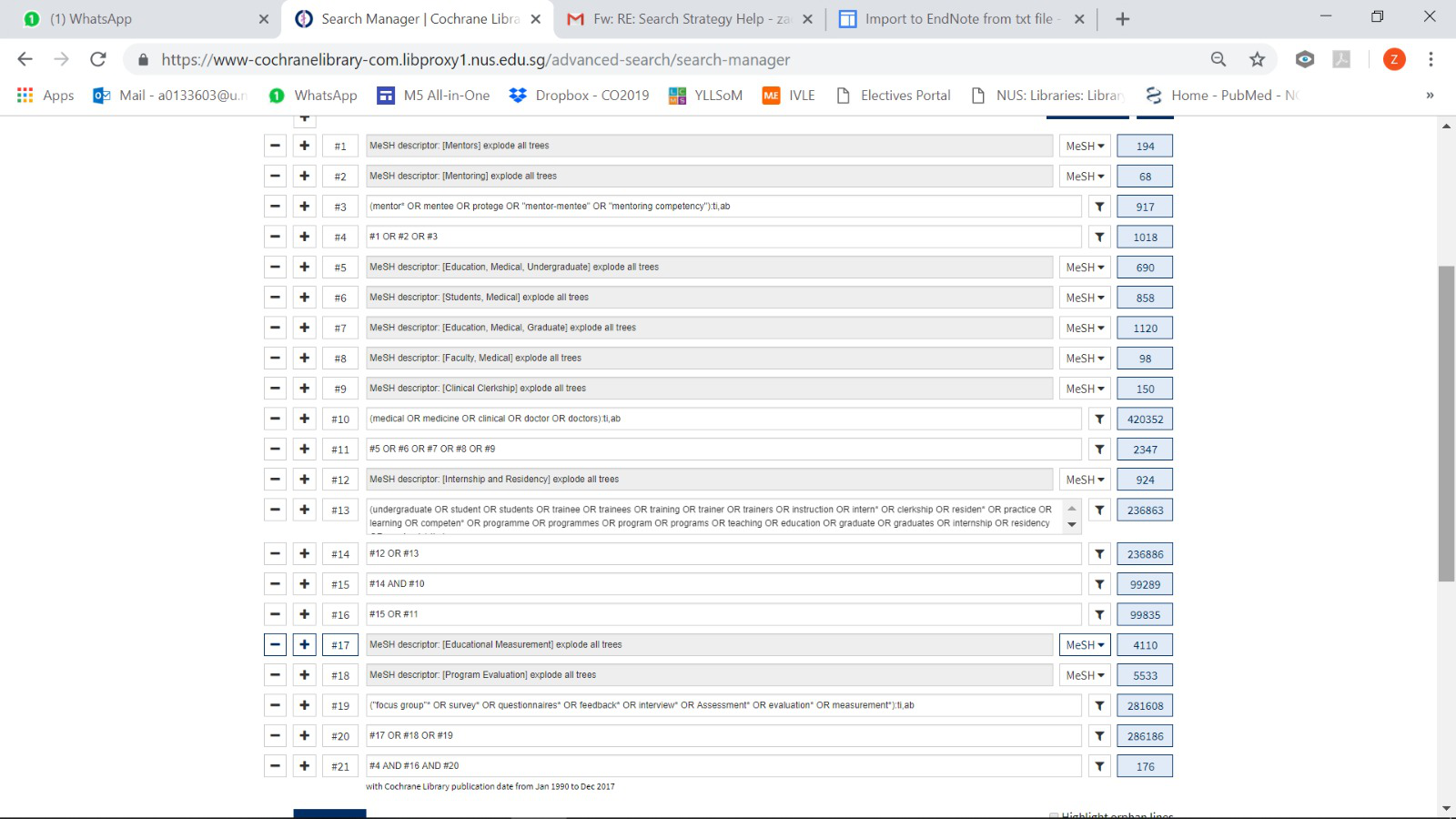


*Cochrane Library search extended to include Jan 2018 to Dec 2019

GreyLit, Web of Science, Open Dissertations (EBSCO), British Education Index (EBSCO)

(((mentors[MeSH Terms] OR mentoring[MeSH Terms] OR mentor*[Title/Abstract] OR mentee[Title/Abstract] OR protege[Title/Abstract] OR “mentor-mentee"[Title/Abstract] OR “mentoring competency”[Title/Abstract])) AND ((“Education, Medical, Undergraduate”[MeSH Terms] OR “Students, Medical”[MeSH Terms] OR “education, medical, graduate”[MeSH Terms] OR “Faculty, Medical”[MeSH Terms] OR “Clinical clerkship”[MeSH Terms]) OR ((medical[Title/ Abstract] OR medicine[Title/Abstract] OR clinical[Title/Abstract] OR doctor[Title/Abstract] OR doctors[Title/Abstract]) AND (undergraduate[Title/Abstract] OR student[Title/Abstract] OR students[Title/Abstract] OR trainee[Title/Abstract] OR trainees[Title/Abstract] OR training[Title/Abstract] OR trainer[Title/Abstract] OR trainers[Title/Abstract] OR instruction[Title/Abstract] OR intern*[Title/Abstract] OR clerkship[Title/ Abstract] OR residen*[Title/Abstract] OR practice[Title/Abstract] OR learning[Title/Abstract] OR competen*[Title/Abstract] OR programme[Title/Abstract] OR programmes[Title/Abstract] OR program[Title/Abstract] OR programs[Title/Abstract] OR teaching[Title/Abstract] OR education[Title/Abstract] OR graduate[Title/Abstract] OR graduates[Title/Abstract] OR internship[Title/Abstract] OR residency[Title/Abstract] OR “internship and residency”[MeSH Terms] OR academic [Title/Abstract])))) AND ("Educational Measurement"[Mesh] OR “Program Evaluation”[Mesh] OR Focus Group*[tiab] OR Survey*[tiab] OR Questionnaires*[tiab] OR feedback*[tiab] OR interview*[tiab] OR Assessment*[tiab] OR Evaluation*[tiab] OR Measurement*[tiab]
