# Supplementary material for: Assessing mentoring: A scoping review of mentoring assessment tools in internal medicine between 1990 and 2019
Source: PLoS One. 2020 May 8;15(5):e0232511. doi: 10.1371/journal.pone.0232511 (PMC7209188; doi:10.1371/journal.pone.0232511)
Supplement: S2 Appendix — (DOCX) [file pone.0232511.s002.docx]

Appendix 2: Summary of included articles

| **No.** | **Paper title** | **Author and year of publication** | **Study location** | **Study type** | **Comparator** | **Duration of study** | **Study population** | **Aims of study** | **Methodology** | **Validity of methodology** | **Outcome measures** | **Important results** |
| --- | --- | --- | --- | --- | --- | --- | --- | --- | --- | --- | --- | --- |
| 1 | Mentoring in Medicine: Introducing a structured programme in a Medical College in Delhi | Ahmed et al 2017 | India | Cross sectional | - | - | Mentee | To evaluate the response to mentoring amongst medical students after introduction of a structured mentorship programme | 10 qns, both closed and open ended | Pre-validated questionnaire, nil elaboration on validation process | Clarity of concept to mentee, duration of being involved in the programme, preferred, mentoring model, comments on intended benefits and those obtained, frequency of meetings, forms of communications used and preferred place of interaction | 1) Lack of common free time is an obstacle to interaction  2) Most of the mentees appreciated the initiative of mentorship 3) The mentorship programme found good acceptance with medical students 4) Students desire to have a role in the allocation of mentors 5) The preferred mentorship model is of one-to-one mentorship |
| 2 | Gastroenterology Faculty and Fellows Identify the Benefits of a Mentoring Program | Borum et al 2015 | US | Cross-sectional | - | - | Both | To evaluate the benefits and challenges of a formal mentoring program in a gastroenterology fellowship program | Nil | Non-validated | Support of professional development, provision of personalized career advice, benefits and challenges of one-on-one mentoring during fellowship training | 1) Benefits include personalized support for professional development and career advice 2) Program fostered trusted relationships, personalized advocacy and improved faculty-fellow comunication 3) Fellows set highter personal goals and improved exam score because of mentor 4) Challenge identified (1): Limited interaction with other faculty because of mentoring assignment 5) Challenge identified (2): there should be a formal process for changing mentors if desire |
| 3 | Effects of Implementing 1-on-1 Mentorship on Development of Medical Knowledge in a Gastroenterology Fellowship Program | Chandler et al 2015 | US | Mentoring program | Gastroenterology training exam (GTE) scores before and after implementation of mentorship program | 1 year | Mentee | To report effects of a 1-on-1 mentorship program on the development of medical knowledge in a university-based, gastroenterology training program | Gastroenterology training exam scores | Non-validated | - | 1) Mentorship is an important component of professional training and has shown to coincide with an increase in the scores on the GTE |
| 4 | Mentorship Programs Can Provide an Opportunity to Enhance Fellowship Education in ACGME Core Competencies | Chandler et al 2015 | US | Mentoring program | Trainee scores on impact of mentoring on behaviours defined by the 6 core ACGME competencies | 1 year | Mentee | To evaluate the effect of a 1-on-1 mentorship program on the six core competencies | Nil description of question number Questions surronded 6 core ACGME compentencies and were grouped into positive or negative/not applicable | Non-validated | Domains of survey: Patient Care, Medical Knowledge, Interpersonal Skills and Communication, Practice-Based Learning and Improbement, System-based Practice, Professionalism | 1) Mentorship had the greatest perceived impact on Patient Care, Medical Knowledge and Interpersonal Skills and Communication 2) With more specific instruction, we believe that 1-on-1 mentorship can also have an impact on Practice-Based Learning, Systems-based Practice and Pro- fessionalism. |
| 5 | Developing senior doctors as mentors: a form of continuing professional development. Report of an initiative to develop a network of senior doctors as mentors: 1994-99 | Connor et al 2000 | UK | Cross-sectional | - | - | Mentor | Evaluation of 4 programs which aimed to trasnfer mentoring skills to everyday medical practice and managing juniors | Focus groups + postal questionnaire | Focus groups were conducted to elicit items for inclusion | 1) Mentor's expectations of program  2) Degree of usefulness of different elements of mentor training 3) Areas of professional development 4) Development of mentoring network for junior and senior doctors | 1) Programmes are highlly valued by particupants, particularly with regard to: being part of a network of senior doctors, developing mentoring skills, and engaging in personal and professional development  2) The most difficult part of the programme was setting up mentoring networks for junior doctors, and reasons included: personal factors, such as levels of confidence in providing mentoring; cultural factors, such as juniors not wishing to be seen to need help, and organizational factors, such as lack of time allocated for mentoring. |
| 6 | Mentored student project for inculcating research skills | Devi et al 2010 | India | Mentoring program | - | - | Mentee | Evaluation of a new mentored student project programme | 16 question close-ended questionnaire | Non-validated | 1) Research skills gained by undertaking projects 2) Guidance received from the mentor | 1) Majority of students felt the program had increased their research skills 2) Type of project and previous research experience were not siginificantly related to improvement in overall skills |
| 7 | Characteristics of mentoring relationships formed by medical students and faculty | Dimitriadis et al 2012 | Germany | Mentoring program | Performance at final secondary-school examinations and Step 1 of the German National Board Examination in medicine | 1 year | Both | Evaluate and characterise mentoring relationships formed between faculty and medical students | 1) Pre-mentoring questionnaire 34 close-ended questions 2) Feedback after every personal meeting 3) Detailed evaluation of program at the end semester (characterize their mentoring relationships, and judge the impact of mentoring on their academic progress)  4) Performance different between student groups at examinations | Modified from Mentorship Profile Questionnaire and Mentorship Effectiveness Scale | 1) Expectations (expectations regarding role of their mentor, mentoring relationship, and topics to discuss with future mentor) 2) Outcomes measures on career planning, research activities, clinical electives, experiences abroad, extra-curricular activities, work-life balance, preparation for exams | 1) Medical students s with strong academic performance as defined by their grades are more likely to participate in formal mentoring programs 2) Mentoring relationships between faculty and medical students are perceived as a mutually satisfying and effective instrument for key issues in medical students’ professional development 3) A mentoring program can thus establish a feedback loop enabling the educational institution to swiftly identify and address issues of medical students. |
| 8 | Mentoring program design and implementation in new medical schools | Fornari et al 2014 | US | Cross-sectional | - | - | Mentor | To evaluate how mentoring programs are being established in new medical schools created since 2006 | 45-item, both close and open ended | Initial questions based on review of literature, then reviewed and revised through series of collaborative discussions among authors | Evaluating how new mentoring programs are established and the variability of their characteristics compared to established mentoring programs | There is no single best practice for the design and execution of mentoring programs More studies are necessary to determine how new mentoring programs evolve as new schools develop and how differences in initial design and implementation affect the quality and outcome of mentoring programs |
| 9 | Mentoring programs for medical students - a  review of the PubMed literature 2000 - 2008 | Frei et al 2010 | Switzerland | Cross-sectional | - | - | Both | To investigate what types of programs exists, what the objectived pursured by such programs are, and what effects are reported | Pubmed Literature search | Not mentioned | Aim of investigation: (1) What types of structured mentoring programs for medical students are reported in scientific medical literature between 2000 - 2008?  (2) What are the objectives pursued by these programs?  (3) What concrete statements, if any, can be identified regarding the effects of mentoring programs?  (4) What additional information is given in scientific literature (2000 - 2008) on different aspects of mentoring for medical students? | Aims of mentoring programs were to provide career counseling, develop professionalism, increase students' interest in research, and support them in their personal growth The personal student-faculty relationship is important in that it helps students to feel that they are benefiting from individual advice and encourages them to give more thought to their career choices. More mentoring programs should be developed, but would need to be rigorously assessed based on evidence of their value in terms of both their impact on the career paths of juniors and their benefit for the mentors Effects of mentoring must be more clearly documented for it to receive more appreciation |
| 10 | A study of satisfaction of medical students on their mentoring programs at one medical school in Korea | Ho et al 2017 | South Korea | Cross-sectional | - | - | Mentee | To investigate the awareness levels of medical students regarding the characteristics of each function within a mentoring program conducted within Kyung Hee University and to ultimately suggest points for reformation | 29-item questionnaire + interviews | Non-validated | Measures career development, psychosocial and friendship functions and subcategories which are composed of specific behaviour indicators that measure each function | 1) Little impact of gender on satisfaction levels 2) Higher the grade in school, the highter the satisfaction levels 3) The higher the number of meetings, the highter the satisfaction levels  4) Average time of meeting and meeting places hardly impact satisfaction levels |
| 11 | Perception of physicians about medical education received during their Nephrology residency training in Peru | Herrera-Añazco et al 2015 | Peru | Cross sectional | - | - | Mentee | To assess the perception of physicians who attend different hospitals and university programs for training of specialists in nephrology | Nil info on number of questions, close-ended questions | Non-validated | 1) Tutoring in Nephrology 2) Clinical training 3) Procedures  4) External rotation 5) Study/research  6) Overall perception | Overall perception of nephrology residency training was considered good; however, areas of tutoring, and academic and research activities on average were deficient. |
| 12 | Preparing to Care for an Aging Population: Medical Student Reflections on Their Clinical Mentors Within a New Geriatrics Curriculum | Farrell et al 2013 | US | Interventional | - | 1 year | Mentee | To analyze medical students’ journals regarding their mentored experiences within a new geriatrics curriculum at a U.S. medical school | Reflective writing/journalling | Not mentioned | 1) the geriatrics content in medical school courses 2) experiences and insights regarding clinical encounters with older persons  3) Occasional third question on that week's particular lecture topic or clinical experiences | 1) Exposure to older adults in a mentored setting may help reverse negative preconceptions about their care 2) the satisfaction of increased contacts may overcome their hesitation in assuming responsibility for managing elders’ complicated medical problems |
| 13 | Students’ perception of mentoring at Bahria University Medical and Dental College, Karachi | Ali et al 2015 | Saudi Arabia | Cross sectional study | - | - | Mentee | To assess the students' perception regarding mentoring at different stages of their studies at a private- sector medical college | 35 close-ended questions | Modified from Mentorship Effectiveness Scale + literature searches and were revised until agreement within panel was attained | 1) 17 questions on personal support 2) 6 questions on career advising 3) 10 questions on Role Modelling and Family 4) 2 questions on Research Collaboration | 1) Students in pre-clinical years (1st/2nd years) rated their mentors higher on role modelling aspects of mentoring (p<0.001) compared to those in the clinical years (3rd/4th years  2) Agreement for personal support had the highest score out of the four categories which was not different among all the four years |
| 14 | A call for mentoring of medical students in the backdrop of integrated cirriculum | Sadiq et al 2013 | Pakistan | Cross sectional study | - | - | Mentee | Conducted for need assessment and to gain students' perspectives regarding establishing a mentoring system | 10 close-ended questions | Non-validated | 1) Assessment of satisfaction level of students towards choice of profession and ability to cope with stress as a reults of academic responsibilities and change to an integrated modular system 2) Awareness of mentoring and presence of informal mentoring in institution 3) Whether environment was conducive for a healthy student faculty interaction | 1) Participants agree to the need of having a formal mentoring program  2) There is lack of training and trend that inhibits communication between faculty and students |
| 15 | A Novel Measure of “Good” Mentoring: Testing Its  Reliability and Validity in Four Academic Health  Centers | Pololi et al 2016 | US | Interventional | Mentoring composite variable representing faculty mentoring experiences as positive, neutral, or inadequate and with other C-Change dimensions of culture. | 1 year | Mentor | To assess mentoring activities experienced by faculty and evaluated evidence for its validity | 6 additional domains of inquiry on top of orginal C-change survey | Modified from original C-Change + literature review + expert consensus | 1. Formulate your career goals 2. Plan how to achieve your career goals 3. Learn the skills needed to succeed in your career goals 4. Find the resources you need 5. Have a sponsor/champion to advance your career or your work 6. Plan how to achieve your personal goals | 1) The refreshed C-change Faculty Survey is a valid instrument to assess mentoring |
| 16 | A structured mentorship program increases residents' satisfaction with mentoring: A two-year cohort study at a multi-hospital internal medicine residency program | Moskowitz et al 2010 | US | Interventional | Intervention arm - residents enrolled in structured mentorship program  Control arm - residents never enrolled in structured mentorship program | 3 years | Mentee | Determine whether a new structured mentorship program improved resident satisfaction with career guidance, compared to previous unstructured mentoring process | 16 closed ended questions | Non-validated | 1) Impact of faculty relationships on career plans 2) Assistance in finding the "right" mentor 3) Help to find a research project suiting resident's career goals | 1) Enrolled residents had increased overall satisfaction with mentoring at their residency program 2) Increased overall satisfaction finding the "right" mentor  3) No different in percieved identifcation of mentor, assistance of faculty to make professional contacts, find a research project, abaliability of faculty mentors, or sufficient contact with an identified mentor |
| 17 | A year of mentoring in academic medicine: Case Report and Qualitative Analysis of Fifteen Hours of Meetings Between a Junior and Senior Faculty Member | Rabatin et al 2004 | US | Interventional | - | 1 year | Both | To address this lack of clarity regarding the components of an effective mentoring relationship, we set out to systematically describe one successful mentoring experience | Qualitative analysis of audiotapes of mentoring session + process journal by mentee | Not mentioned | 3 authors independently coded the transcript for themes and used an iterative consensus-building process to identify a common understanding of salient aspects of mentoring process and important themes within mentoring | 4 important aspects of mentoring 1) Mentoring meetings (structure, refinment of goals)  2) Themes 3) Interactive and personal qualities 4) Outcomes |
| 18 | Faculty Mentors for Interaction over Four Years | Payer et al 1996 | US | Cross-sectional | - | - | Mentee | To provide continuity cross program through involvement of faculty at the administrative, evaluation of the entire program | Nil | Non-validated | Evluation of mentor's roles in the cirriculum, including numberic ratings and narratives, avaliability for student contact, value of actual teaching time, value of performance feedback, receptiveness to student questions, willingness to admit uncertainty, level of enthusiasm | 1) Mentors have become familiar with personal, academic, professional development of students in the ILT  2) Participation in all of administrative and committee activities of iLT has enabled them to provide continuity to the planning, implementation, evaluation of ILT |
| 19 | Impact of a formal mentoring program on academic promotion of Department of Medicine faculty: A comparative study | Morrison et al 2014 | Canada | Interventional | Comparisons of time to promotion before and after implementation of a formal mentoring program, and between mentored and non-mentored faculty | 12 years | Mentee | To evaluate the impact of a formal mentoring program on time to academic promotion and differences in gender-based outcomes. | Promotional data over 12 years | Not mentioned | The primary outcome measure was the number of years to promotion from one faculty level to another aggregated by, and matched on, academic job descriptions, gender, age at appointment, medical specialty, and rank. These measures were compared between the faculty appointed prior to 2003 and those appointed in 2003 or after. A secondary a priori comparison included the mean number of years to promotion from one faculty level to another between faculty members with a mentor and those without a mentor, regardless of year of appointment. | 1) Mentoring was a powerful predictor of promotion, regardless of the year of appointment and likely benefited both genders equally. University resource allocation in support of mentoring appears to accelerate faculty advancement  2) Gender effects were statistically insignificant. Post hoc analyses of time to promotion suggested that observed differences are not attributable to temporal effects, but rather assignment to a mentor |
| 20 | Lessons in mentoring | Selwa 2003 | US | Cross-sectional | - | - | Mentee | To report the results of a survey of former traines on the unique mentoring style of Dr Sid Gilman | 10 close-ended questions + 2 open ended questions on other mentors and additional comments on Dr Gilman's mentoring | Non-validated | (1) I consider Dr. Gilman to be one of my mentors in Neurology (trainees who answered 1 or 2 to this question did not consider Dr. Gilman to be a mentor and were deleted from the survey). (2) Dr. Gilman took a personal interest in my growth and development. (3) Dr. Gilman supported my clinical interests. (4) Dr. Gilman supported my academic interests. (5) Dr. Gilman supported my efforts to begin new endeavors with as yet undetermined value (financial or academic) to the department. (6) Dr. Gilman’s direction of the teaching program enhanced my training. (7) Dr. Gilman’s personal teaching enhanced my training. (8) Dr. Gilman positively influenced my long-term career choices. (9) I consider Dr. Gilman to be a very effective mentor. (10) How many years did you spend at Michigan? | The ideal mentor possesses “a generous measure of intellectual ability, integrity, both personal and social, honesty so obvious and crystal that someone has called it ‘transparent integrity,’ a passion for truth, a motivation that makes social sense, emotional stability, the habit of working under his own drive, a capacity for growth, curiosity, the ability to respond with imagination and creativity to new or challenging situations, tolerance of the differences among people and reverence for life, personality and the dignity of man.” This is an excellent description of the mentoring provided by Dr. Sid Gilman during his 26 years as Chair at the University of Michigan. |
| 21 | Mentoring among Pakistani postgraduate resident doctors | Sheikh et al 2016 | US | Cross-sectional | - | - | Both | To gauge the quality of mentorship at King Edward Medical University and discover factors responsible for satisfaction or dissatisfaction of mentors and mentees | Mentees: 9 close-ended questions + 5 open ended Mentors: 6 close-ended questions + 4 openended | Literature review + online focus group + consultancy with experts + pretest on a small sample | 1) Frequency of meetings 2) Goald and expectations 3) Career advice 4) Overall satisfaction 5) Mentoring environment 6) Problems with mentoring | 1) Mentees who had a defined set of goals and expectations with their mentors were found to be more satisfied than those without 2) satisfaction level of mentees greatly depended on receiving a roadmap towards advancing one’s career 3) Significant association between satisfaction level and gender 4) There is a lack of opportunities for effective mentorship at King Edward Medical University. Adequate training of mentors and mentees to adopt strategies for a better career outcome of young doctors should be in place |
| 22 | Mentoring Matters Mentoring and Career Preparation in Internal Medicine Residency Training | Ramanan et al 2006 | US | Cross-sectional | - | - | Mentee | To describe mentoring relationships among internal medicine residents, and to examine the relationship between mentoring and perceived career preparation | 58 close-ended questions | Literature review + pilot testing + respondent interviews post-pilot | 1) Examine importance that residents place on mentoring during residency 2) Whether residents report that their residency program encouraged mentoring 3) Satisfaction with global and specific qualities of mentoring 4) Confidence in career decisions 5) Support when faced with uncertainty 6) Barriers to developing a mentoring relationship 7) Qualities associated with satisfying mentorship 8) How mentoring relationships were established and maintained | 1) Positive relationship between mentoring and perceived career preparation 2) Relative lack of mentoring among interns and underrepresented minority residents |
| 23 | “Mentorship” a stride towards maintenance of medical student’s well being | Rehman et al 2014 | Pakistan | Cross-sectional + interventional | - | - | Mentee | To evaluate the effectiveness of mentorship programme with respect to emotional wellness of medical students | 11 close-ended | Non-validated | 1) Emotional wellness 2) Coping strategies | The mentoring program provided emotional support, minimised the stress, limited consultation by psychologists or psychiatrists and reduced reservations of parents and students of first year medical education |
| 24 | ‘‘Having the Right Chemistry’’: A Qualitative Study of Mentoring in Academic Medicine | Jackson et al 2003 | US | Cross-sectional | - | - | Mentee | To develop a deeper understanding of mentoring by exploring lived experiences of academic medicine faculty members | Phone interview | Literature review + focus group content analysis | 1) Participant's demographics 2) Factors hindering career progression 3) Mentor's academic and psychosocial activities  4) Matching 5) Characteristics of prized mentoring relationships 6) The academic parent or coach 7) Supporting/enabling actions | Having a mentor is critical to having a successful career in academic medicine. Mentees need to be diligent in seeking out these relationships and institutions need to encourage and value the work of mentors. Participants without formalized mentoring relationships should look to peers and colleagues for assistance in navigating the academic system |
| 25 | “A Good Career Choice for Women”: Female Medical Students’ Mentoring Experiences: A Multi-Institutional Qualitative Study | Levine et al 2013 | US | Cross-sectional | - | - | Mentee | To explore the role gender plays in the mentoring experiences of female medical students | Focus groups | Literature review + iterative process to finalize focus group guide | 10 prompts used to guide discussion covering  1) factors of successfu/unsuccessful mentoring/advising relationship  2) barriers to developing a successful mentoring relationship 3) Different kinds of advisors/mentors  4) Times/situations where a female/male advisor is specifically sought out for 5) Expectations 6) Gender differences in interactions | The authors identified four major themes:  (1) Optimal mentoring relationships are highly relational. Students emphasized shared values, trust, and a personal connection in describing ideal mentoring relationships  (2) Relational mentoring is more important than gender concordance. Students identified a desire for access to female mentors but stated that when a mentor and mentee developed a personal connection the gender of the mentor was less important  (3) Gender-based assumptions and stereotypes affect mentoring relationships. Students described gender based assumptions and expectations for themselves and their mentors  (4) Gender-based power dynamics influence students’ thinking about mentoring. Students stated that they were concerned about how their mentors might perceive their professional decisions because of their gender, which influenced what they disclosed to male mentors and mentors in positions of power |
| 26 | An Initiative in Mentoring to Promote Residents’ and Faculty Members’ Careers | Levy et al 2004 | US | Mentoring program | - | - | Mentor | To report the feedback received from a survey of the 2002–03 medical housestaff about their experiences with the initiation of a mentoring program | 8 close-ended questions | Non-validated | 1) Is it important that the Department of Medicine assigns you a faculty mentor? 2) Do you communicate with your mentor by phone or e-mail? 3) Over the last year, has the frequency of meetings with your faculty mentor been okay with you?  4) Has your faculty mentor been helpful? 5) Has your faculty mentor been available? 6) Have you developed unassigned mentoring relationships? 7) Are you interested in switching to a different faculty mentor? 8) My mentor gets to know me as an individual. | The formal mentoring program has ensured that all trainees are provided with a mentor, which has facilitated faculty– housestaff interactions and increased recognition of faculty contributions to mentoring |
| 27 | Being a mentor for undergraduate medical students enhances personal and professional development | Stenfors-Hayes et al 2010 | Sweden | Cross sectional study | - | - | Mentor | This study aims to evaluate an undergraduate mentor programme from the mentors’ perspective, focusing particularly on the effect of mentorship, the relationships between mentoring and teaching and the mentors’ perceived professional and personal development. | 12 closed-ended questions + 2-3 open-ended + 30-45 mins individual interviews | New tool, developed from thematic analysis, focus groups, extensive literature review | 12 closed-ended questions + 2-3 open-ended + 30-45 mins individual interviews  Domains: Topics of discussions in meetings, description of what it was like to be a mentor, what do you think you do in your role as a mentor, degree of personal/professional development from being a mentor, | 1) Being a mentor increased interest in teaching and reflections regarding own values and work practices 2) Majority of respondents developing teaching as result of mentorship and improved relations with students 3) Training for mentor is beneficial  4) Unclear definition of professional development exisits 5) Difficulty illustrating essence of benefits of benefits of being a mentor by many respondents |
| 28 | Development and validation of the Medical Student Scholar-Ideal Mentor Scale (MSS-IMS) | Sozio et al 2017 | USA | Mentoring program | - | - | Mentee | We sought to modify and validate a mentoring scale for use in medical student research experiences. | 11qns, closed ended | Non-validated | 11-item scale  Domains: 1) Integrity 2) Guidance + 3) Student sense of ownership over project + overall satisfaction with mentoring | 1) 8 items correlated with 4 student outcomes (mentor satisfaction, liklihood of future research, abstract submission to national meetings, SC faculty rating of student projects)  2) Revised scale can be used to advance medical students scholarship |
| 29 | Differential mentorship for medical students: Development, implementation and initial evaluation | Thorndyke et al 2006 | Germany | Mentoring program | - | - | Both | The aim of this study was to develop a uniquely tailored mentoring program for medical students and evaluate the success of implementation. | Open and closed ended | Non-validated | Domains: Publicity + acceptance of programme within target group | Satisfying response rate in terms of awareness and increasing participation |
| 30 | Empowering Junior Faculty: Penn State’s  Faculty Development and Mentoring Program | Kurre et al 2012 | USA | Mentoring program | - | - | Both | Effectiveness of their program | 20-item post course survey, 11 item self-assessment (before and after programe), the rest are unknown: open and closed ended | Non-validated | 20-item post course survey, 11 item self-assessment (before and after programe), the rest are unknown  Domains of 20-item post course survey: Overall assessment of program perceived impact on participants careers, effectiveness of mentoring program  Domains of self-assessment: self-perception of their own abilities | 1) Participants evaluated the program highly  2) Demonstrated increases in perceptions of own abilities 3) Completed tasks important to advancement of careers  4) Participants better prepared to advance academic careers 5) 6 strategies/recommendations for creating high-impact faculty development programs |
| 31 | Functional Mentoring: A Practical Approach With Multilevel Outcomes | Thorndyke et al 2008 | USA | Mentoring program | - | - | Both | To address this issue, the authors advance the framework of functional mentoring combined with measurement of outcomes at multiple levels. | 18 question end of program questionnaire, multiple open-ended questions, 13 question mentor questionnaire for program, 5 question longitudinal questionnaire, skill development surveys: Open and closed | Non-validated | 18 question end of program questionnaire, multiple open-ended questions, 13 question mentor questionnaire for program, 5 question longitudinal questionnaire, skill development surveys  Domains of end-of-program questionnaire: Overall satisfaction, demographics, structure and framework, value of mentoring, impact of relationship, impact of project  Domains of mentor questionnaire: Same  Longitudinal study: Presence of continued relationships | 1) Respondents were highly satisfied with pairings 2) 85% of junior faculty believed mentors had significant effect on their projects  3) Significant enhancement of skills through program  4) Mutli-level validation of program success proves that "functional mentoring" is a viable approach |
| 32 | Introducing Mentoring to 1st­year Medical Students of a Private Medical College in North India: A Pilot Study | Kukreija et al 2017 | India | Mentoring program | - | - | Both | The aim of this study is to introduce and to assess the perception of mentors and mentees on mentorship program. | 1) Mentee - 23 Close-ended, 3 open ended 2) Mentor - 10 Close-ended, 5 open ended  Open and closed ended | Non-validated | 1) Mentee - 23 Close-ended, 3 open ended 2) Mentor - 10 Close-ended, 5 open ended  Domains: Avaliability, communication, support, resources and ideas for studies, willingness of volunteering for future projects Open-ended questions:  1) What is going well in the mentorship program  2) What challenges have you confronted in the mentorship program? 3) What are the changes you idenfitied in youself as a result of this mentorship program 4) Any other suggestions 5) Overall evaluation of mentorship program | 1) Mentees felt supported in terms of personal development, motivated to improve academic performance  2) Mentors and mentees felt program improbed communication between them 3) Mentors had feeling of self-satisfaction 4) Teaching skills of mentors improved through program 5) Program built a better student-teacher relationship |
| 33 | Issues in the Mentor–Mentee Relationship in Academic Medicine: A Qualitative Study | Straus et al 2009 | Canada | Cross sectional study | - | No mention | Both | To explore the phenomenon of the mentor–mentee relationship and to characterize this relationship among people who have obtained early career support from a government funding agency, in order to facilitate the development of future mentorship programs. | 1) Mentees - 14 open-ended questions  2) Mentors - 7 open-ended questions  Closed ended | Non-validated | 1) Mentees - 14 open-ended questions  2) Mentors - 7 open-ended questions Mentee developed domains of inquiry:  1) What is the experience of mentorship that you have received? 2) How and when did you identify your mentor (s)? 3) Were there any barriers or facilitators to identifying a mentor? 4) How often do you meet with your mentor 5) What do you receive from your mentor? What do you discuss with your mentor? What is the function of your mentor? 6) What do you perceive as the elemtns of successful mentoring relationship? Of a failed mentoring relationship? 7) Do you mentor anyone? 8) Does gender play a role in the mentor-mentee relationship? Should the mentor and mentee be the same gender? 9) Can you identify any relevant materials from your organization on formal mentorship programs? 10) What was your mentor's role in preparing your application for funding from the Alberta Heritage Foundation for Medical Research? What was your experience of mentorship in this process?  11) What mentorship strategies would you recommend be implemented (if any) at your organization? At funding agencies?  12) Were there any barriers to receiving mentorship? 13) If any barriers were identified, what could be done to overcome these barriers? 14) Should mentors be matched on gender, culture or other factors?  Mentors domains of inquiry:  1) What is the experience of mentorship that you have received?  2) How would you characterize their relationship with your mentee? 3) What do you perceive as the elements of a successful mentoring relationship? Of a failed mentoring relationship? 4) In your role as mentor, what do you aim to do? 5) Does gender play a role in the mentor-mentee relationship? 6) Can you identify any relevant material from your organization on formal mentorship programs? 7) What was your role in your mentee's application for Alberta Heritage Foundation for Medical Research funding? | 1) 6 themes identified through transcript analysis (overall experience with mentorship, experience of being assigned a mentor versus self-identification of a mentor, roles of a mentor, characteristics of a good mentoring relationship, barriers to mentorship, and possible mentorship strategies)  2) Severeal threats to a failed mentorship program  3) Mentors should be self-identified and not formally assigned  4) Failures in mentoring include blurred lines between intellectual property of mentor and mentee  5) Study correlates with literature on role of mentor |
| 34 | Longitudinal mentorship to support the development of medical students’ future  professional role: a qualitative study | Kalen et al 2015 | Sweden | Mentoring program | - | - | Mentee | The aim of this qualitative study is to explore how formal and longitudinal mentoring can contribute to medical students’ professional development. | Open ended | No mention | No information on design of question guide. Content covered mentioned as experiences of different parts of the workshop days, development and mentorship experiences | 3 Major themes 1) Integrating oneself with one's future role as a physician 2) Experiencing clinial reality with the mentor creates incentives to learn 3) Towards understanding the professional competence of a physician   Mentoring focusing on non-medical skills can help medical students to integrate the professional role with their own personalities and promote their understanding of the wholeness of professional competence in the process of becoming a physician |
| 35 | Mentoring at the University of Pennsylvania: Results of a Faculty Survey | Wasserstein et al 2007 | USA | Mentoring program | - | - | Mentee | To explore multiple aspects of mentoring at an academic medical center in relation to faculty rank, track, and gender. | 17qns, Closed ended | Modified from another survey (non-validated) | 2 groups of closed-ended questions 1) Formation of mentoring relationship, no of mentors, characteristics of primary mentor, satisfaction with mentoring 2) Specific assistance (giving advice - 10 items, providing opportunities - 7 items) | 1) Most assistant/associate professors had a mentor  2) satisfaction with mentoring was correlated with no of types of mentoring received, job satisfaction, meeting frequency and expectation of leaving university within 5 years  3) Significant predictors of higher overall job satisfaction were associate rank, 10-point mentoring satisfaction rating and no of mentors 4) Rare study that examines mentoring relationships in associate professors 5 conclusions stand out for the study |
| 36 | Mentoring for NHS doctors: perceived benefits across the personal–professional interface | Steven et al 2008 | UK | Cross sectional study | - | Not mentioned | Both | To investigate NHS doctors’ perceived benefits of being involved in mentoring schemes and to explore the overlaps and relationships between areas of benefit. | Open ended | New tool (developed from literature review and discussions at Doctor's forum) | Domains include problem solving, patient care, job satisfaction, relationships, performance, personal wellbeing, leadership, personal and professional development | This paper is secondary analysis of primary study, with 3 overarching themes developed 1) Professional practice  2) Personal well-being 3) Development  On top of that, benefits go beyond professional realm and crosses personal-professional interface Problem solving and change management appear to be key processes underpinning raft of personal and professional benefits reported |
| 37 | Mentoring undergraduate medical students: Experience  from Bahria University Karachi | Usmani et al 2011 | Pakistan | Mentoring program | - | - | Mentors | To explore the perceptions and the effects on mentors regarding mentoring medical students at Bahria University Medical and Dental College, Karachi, Pakistan. | Open and closed ended | Non-validated | General mentoring questions to the mentor posed, some about the usefulness of mentoring to both mentor and mentee, interest in mentee, trust in the relationship, feedback | 1) majority of the mentors rate themselves as "good" or "satisfactory"mentors and felt that their mentees showed good improvement academically due to these sessions. 2) The perception of mentors about the structured mentoring programme subjectively appears to be a promising strategy for young medical students.  3) Objective assessment of these mentors is needed. |
| 38 | Mentors in Graduate Medical Education at the Medical College of Wisconsin | Kirsling et al 1990 | USA | Presence of mentors | - | - | Mentors - Senior faculty members | Determine perceptions of the extent and benefits of mentor relationships between faculty and residents | Closed ended | Non-validated | The authors designed 2 questionnaires; 1st questionnaire dealt with faculty member's definitions of mentor relationship and perceptions of feasibility of mentor activity with residents, 2nd questionnaire was only for those who had a mentor or had been a mentoring- regarding age and gender differences and benefits of the relationship. | 1) 78% thought mentor activity was feasible in their own departments;  2) only 18% felt that a majority of residents in their departments had a mentor.  3) Positive benefits to mentoring were significant (career advancement, personal development, dealing with stress)  4) The mentor relationship appears to have significant benefits for the medical trainee and should be promoted |
| 39 | Mentorship in Academic General Internal Medicine  Results of a Survey of Mentors | Luckhaupt et al 2005 | USA | Cross sectional study | - | - | Mentors | To describe current trends in mentorship in general Internal Medicine (GIM). | Open and closed ended | Non-validated | We developed a 25-item survey, with questions based on mentorship issues discussed in the literature and on the authors’ experience. The final survey contained sections about funding for mentorship, multiple mentees, comentorship, long-distance mentorship, and social/professional boundaries. | 1) Compared with mentors without funding, mentors with funding had more current mentees.  2) Full professors had more current mentees than associate or assistant professors. 3) Mentors in GIM appear to be close to their mentorship capacity, and the majority lack funding for mentorship. Comentoring and long-distance mentoring are common. |
| 40 | Perceptions of a faculty mentorship programme | Miedzinski et al 2009 | Canada | Mentoring program | - | - | Both | To facilitate the relationship, a web-based Mentorship Information Kit and a mentorship workshop were offered. | Closed ended | Non-validated | Both groups were asked to evaluate the perceived impact of the relationship on assumed measures of effectiveness, including research funding and publication success, local and national networking, integration into the department, and enhanced life balance. | 1) Significant correlations were noted between the mentee’s assessment of adequacy of meeting frequency and perceived mentor helpfulness. 2) In general, scientific mentoring pairs perceived a greater impact on academic success than academic pairs.  3) Although the overall assessment of the programme was positive, it was difficult to tease out the specific aspects of academia enhanced by the programme, which made it difficult to characterise success. |
| 41 | More mentoring needed? A cross-sectional study of mentoring programs for medical students in Germany | Meinel et al 2011 | Germany | Cross sectional study | - | June to October 2009 | Mentors - Deans in medical education faculty (People who organised the mentoring program) | We conducted this study to survey all medical schools in Germany regarding the prevalence of mentoring programs for medical students as well as the characteristics, goals and effectiveness of these programs. | Total 34; in multiple choice (17), numeric (7) and free-text (10) format, open and closed ended | Non-validated | We developed a questionnaire to assess key characteristics of mentoring programs: the advocated mentoring model, the number of participating mentees and mentors, funding and staff, and characteristics of mentees and mentors (e.g., level of training). In addition, the survey characterized the mentee-mentor relationship regarding the frequency of meetings, forms of communication, incentives for mentors, the mode of matching mentors and mentees, and results of program evaluations. Furthermore, participants were asked to characterize the aims of their programs. | 1) 15 programs (68%) reported conducting evaluations on a regular basis.  2) Two programs reported that results had been submitted for publication in a scientific journal.  3) Evaluations are performed through online or paper surveys, interviews or feedback meetings.  4) Most evaluations focused on mentee and mentor satisfaction corresponding to Kirkpatrick’s level 1. |
| 42 | Redesigning a clinical mentoring program for improved outcomes in the clinical training of clerks | Lin et al 2015 | China | Mentoring program | - | - | Mentees - 5th year medical students | This study assessed the effect of a redesigned clinical mentoring program from the perspective of clerks. The objective was to assess the benefits of the redesigned program and identify potential improvements. | 15qns, closed ended | Modified Scandura and Ragins | The 15-item mentorship scale of Scandura and Ragins, which encompasses mentoring functions such as psychosocial support, career development, and role modeling was adapted by adding the phrase ‘in your clerkship’ before each question item. The scale instrument covered the functions of mentoring from a whole-person perspective that were integrated into the redesigned program | 1) The results revealed that the redesigned mentoring program improved the mentors’ performance over time for most evaluated items regarding professional development and personal support provided to the mentees. |
| 43 | Relating Mentor Type and Mentoring Behaviors to Academic Medicine Faculty Satisfaction and Productivity at One Medical School | Shollen et al 2014 | USA | Nil | - | - | Mentee - Full time faculty | To examine relationships among having formal and informal mentors, mentoring behaviors, and satisfaction and productivity for academic medicine faculty. | 72qns, closed ended | Adapted from John Hopkins University School of Medicine | Questionnaire with selected variables to operationalize the constructs of interest, as described below (role model, career and networking, advice, advocate, critique, work-life balance). | 1) Informal mentoring was more important for satisfaction, and formal mentoring was more important for productivity. 2) Regardless of mentor type, the 14 mentoring behaviors examined related more to satisfaction than productivity |
| 44 | Students’ views of mentoring at Bahria  University Medical and Dental College | Usmani et al 2016 | Pakistan | Mixed methods | - | - | Mentees: students | To explore the mentoring program on a subset of Pakistani medical students in a private medical college. | Open and closed ended | Non-validated | Questionnaire about importance of mentoring for students, development of personality as a result of mentoring, issues with confidentiality | 1) Analysis of the result showed that majority of the students considered mentoring program a beneficial tool for their academic and non-academic lives. 2) The medical students appreciated the presence of a mentor during thick and thin; they have also accepted that it is due to the presence of this guide that they are able to continue with their difficult studies in these difficult times. |
| 45 | The core of mentorship: medical students’ experiences  of one-to-one mentoring in a clinical environment | Kalen et al 2011 | Sweden | Inductive content analysis and mentoring program | - | - | Mentees: students | This study aimed to get a deeper understanding of the meaning of mentorship seen from the perspective of undergraduate medical students. | Open ended | Not mentioned | Question areas were the contents of the students’ and their mentors’ conversations, their relationship, the meaning of the mentorship and the students’ general views on mentoring. | 1) Having a mentor gave a  i) sense of security  ii) constituted a ‘free zone’ alongside the undergraduate programme.  iii) It gave hope about the future and increased motivation. |
| 46 | The impact of mentoring during postgraduate training on doctors’ career success | Stamm et al 2011 | Switzerland | Prospective Study | - | - | Mentees - Graduates | This study made use of a longitudinal design to investigate the impact of mentoring during postgraduate specialist training on the career success of doctors. | Closed ended | Adopted from Mentoring Support Questionnaire, Objective and Subjective Career Success tools | Mentoring Support Questionnaire: This questionnaire comprises five scales for, respectively, ‘sponsorship and networking’, ‘career planning’, ‘coaching’, ‘emotional support’ and ‘role model’. | 1) Both having a mentor and having career support from the development network were significant predictors of both objective and subjective career success.  2) This study confirmed the positive impact of mentoring on career success in a cohort of Swiss doctors in a longitudinal design. |
| 47 | The Importance of Mentoring in Pulmonary and Critical Care Fellowship | Nelson et al 2014 | USA | Nil | - | - | Mentee - Current faculty and fellows | The purpose of this study was to investigate the current state of mentorship within the PCCM fellowship and identify any perceived barriers and characteristics needed to help promote effective mentorship. | Closed ended | Non-validated | Surveys covered benefits to mentorship: career progression, leadership, research. Others include influence of career choice, initiation of mentorship, barriers to mentoring, areas for focus of mentoring relationship | 1) mentorship was important in the field of medicine and careers.  2) The greatest benefits to mentorship:  i - Leadership,  ii- research,  iii - career promotion  iv - advancing in state and national societies.  3) benefit was perceived in developing humanistic qualities and teamwork strategies. |
| 48 | The Munich-Evaluation-of-Mentoring-Questionnaire  (MEMeQ) – a novel instrument for evaluating protégés’ satisfaction with mentoring relationships in medical education | Schäfer et al 2015 | Germany | Mentoring program | - | - | Mentees | The aim of this study was to develop a feasible instrument to measure the satisfaction with mentoring relationships. | 7qns, both open and closed | Non-validated | First part: six items based on the five themes of the ideal qualities of mentorship, which emerged from the analysis of Cho et al.: 1) admirable characteristics of mentors (including enthusiasm, compassion, and selflessness), 2) how mentors serve as career guides, 3) strength of time commitments (regular, frequent, and high-quality meetings), 4) support for mentee’s personal/professional balance, and 5) leaving behind a legacy of mentoring. Second Part: The 2nd part of the MEMeQ deals with the content aspect of mentoring relationships (CAM). includes protégés’ perceptions of instrumental support behaviors considering individual areas of interest and goals. We adapted the core concept of the Schedule for Meaning in Life Evaluation (SMiLE) and transferred it to the mentoring setting. | 1) The two scores of the questionnaire correlated closely with the overall satisfaction regarding mentoring relationships.  2) The authors propose MEMeQ as a reliable, valid and flexible instrument for measuring the weighted satisfaction of protégés with their individual mentoring relationship in medical education. |
| 49 | The role of mentoring in academic career progression:  a cross-sectional survey of the Academy of Medical  Sciences mentoring scheme | Iversen et al 2014 | UK | Mixed methods | - | 27 Oct 2010 - 13 Dec 2010 | Mentees | To describe a successful mentoring scheme designed for mid-career clinician scientists and to examine factors associated with mentee report of positive career impact. | 40qns, open and closed ended | Non-validated | Selection of mentors, experience of mentorship (communciation, role modeling, skill development), benefits to mentorship (work-life balance, career progression, confidence, research), relationship (mutual understanding, setting of expectations, time with mentor), logistics (time spent in mentoring session, initial contact, contact frequency) | 1) Mentoring success was determined by  i - reasons for selection,  ii - mentee characteristics,  iii - experience  iv - skills of the mentor and the quality of the relationship. |
| 50 | Development and initial validation of a dual-purpose questionnaire capturing mentors’ and mentees’ perceptions and expectations of the mentoring process | Heeneman and de Grave 2019 | Netherlands | Mixed methods | - | - | Mentors and Mentees | To design and validate a dual-purpose questionnaire capturing both the mentor and mentee perceptions on the actual and preferred mentoring functions | 38 items, Likert-scale questions, 1 open-ended question | Validated | Self-direction of learning and reflection (Scale 1), guidance of behavioural change (Scale 4), addressing personal issues and professional identity development (Scale 3 and 5) and how the mentor and mentee presents oneself in the mentoring relationship (Scale 2). | 1) Mentors and mentees perceived comparable situations as critical for an effective mentoring process, such as mentor presence and guidance of reflection, although there was also evidence of gaps, such as perception of cultural issues.  2) By comparison of the mentee groups in the different years of the program, the dynamic or evolving nature of the mentor process became evident, mentees experienced more emphasis by the mentor on reflection (Scale 1), at a constant level of mentor presence (Scale 2). |
| 51 | The Medical Oncology resident mentor: situation and workload | Elez et al 2018 | Spain | Quantitative | - | - | Mentors and non-mentoring professionals in Medical Oncology | To highlight the significant workload and unrecognized dedication that being a MO mentor entails, as well as the bearing it has on the quality of teaching units and their accreditation. | 49 closed-ended questions grouped into 6 blocks | Non-validated | 6 blocks of questions including: general data, organization, functions and time of dedication, training needs, recognition, and research | 1) Of the MO mentors, 90% stated that they did not have enough time to perform their mentoring duties. An estimated 172 h/year on average was dedicated to mentoring, which represents 10.1% of the total time. MO mentors dedicate an average of 6.9 h/month to these duties outside their workday.  2) Forty-five percent of the mentors feel that their role is scantly recognized, if at all. |
| 52 | The association between mentoring and training outcomes in junior doctors in medicine: an observational study | Ong et al 2018 | UK | Mixed methods | Pass rates of the various components of the Membership of the Royal College of Physicians (MRCP) (UK) examination (MRCP Part 1, MRCP Part 2 Written and MRCP Part 2 PACES), pass rates at the Annual Review of Competency Progression (ARCP), Significant events/ Clinical incidents/Complaints | 2015 - 2017 | Mentees | To determine quantitatively if a positive association exists between the mentoring of junior doctors and better training outcomes in postgraduate medical training within the UK. | 14 binary, non-Likert questions and 1 open question, "Did mentoring help your career progression?" | Non-validated | 1) MRCP Part 1, Part 2 (Written), Part 2 (Theory) 2) Annual Review of Competency Progression Outcome 3) Significant events, clinical incidents, or complaints 4) Trainee's perception of confidence, career progression, mentored experience | 1) A positive association is observed between the mentoring of core medical trainees and better training outcomes. 2) Mentored trainees reported higher confidence and career progression. |
| 53 | The impact of active mentorship: results from a survey of faculty in the Department of Medicine at Massachusetts General Hospital | Walensky et al 2018 | UK | Quantitative | - | 1 June 2016 - 30 June 2016 | Mentors and mentees, who are faculty in Department of Medicine | To assess mentorship experiences among the faculty of a large academic department of medicine and to examine how those experiences relate to academic advancement and job satisfaction. | 12-page survey with 5-point Likert scale questions | Non-validated | 1) Personal demographics: such as gender, race, ethnicity, and number of dependent children (< 21 years)  2) 10 areas of assessment ranging from: personal and professional characteristics; job satisfaction; mentoring; and prospects for promotion; assistance they received in review of scientific work or grants to provision of opportunities for career advancement to advice about work/family balance, etc | 1) A majority of faculty survey respondents had mentorship, though not all of it of high caliber.  2) Because quality mentorship significantly and substantially impacts both academic progress and job satisfaction, efforts devoted to improve the adoption and the quality of mentorship should be prioritized. |
| 54 | Barriers and Facilitators of Mentoring for Trainees and Early Career Investigators in Rheumatology Research: Current State, Identification of Needs, and Road Map to an Inter-Institutional Adult Rheumatology Mentoring Program | Ogdie et al 2018 | USA | Qualitative | - | June 2015 | Rheumatology fellows, junior faculty, and mentors | To determine perceived barriers and facilitators to effective mentoring for early career rheumatology investigators and to develop a framework for an inter-institutional mentoring program. | Focus groups or interviews | Non-validated | 1) Themes: mentor-mentee relationship, barriers, and facilitators of a productive relationship | 1) Attributes of the ideal mentee-mentor relationship included communication, accessibility, regular meetings, shared interests, aligned goals, and mutual respect. 2) The mentee should be proactive, efficient, engaged, committed, focused, accountable, and respectful of the mentor’s time. The mentor should support/promote the mentee, shape the mentee’s goals and career plan, address day-to-day questions, provide critical feedback, be available, and have team leadership skills.  3) Barriers included difficulty with career path navigation, gaining independence, internal competition, authorship, time demands, funding, and work-life balance.  4) Facilitators of a successful relationship included having a diverse network of mentors filling different roles, mentor-mentee relationship management, and confidence.  5) Primary uses of an inter-institutional mentoring program were career development planning and oversight, goal-setting, and networking. |
