# Supplementary material for: Assessing mentoring: A scoping review of mentoring assessment tools in internal medicine between 1990 and 2019
Source: PLoS One. 2020 May 8;15(5):e0232511. doi: 10.1371/journal.pone.0232511 (PMC7209188; doi:10.1371/journal.pone.0232511)
Supplement: S1 Table — (DOCX) [file pone.0232511.s004.docx]

**S1 Table. Assessment methods**

| Type of study | Quantitative | 27 (50%) | [2, 36, 83, 87, 90, 91, 94, 95, 99, 100, 103-106, 108-111, 113-116, 120, 121, 131-133] |
| --- | --- | --- | --- |
|  | Qualitative | 8 (15%) | [11, 85, 88, 98, 101, 112, 134, 135] |
|  | Quantitative and Qualitative | 19 (35%) | [8, 78-82, 84, 86, 89, 92, 93, 96, 97, 102, 107, 117-119, 122] |
| Method of collecting data | Questionnaires | 19 (35%) | [2, 36, 78, 83, 84, 87, 92, 94, 95, 97, 100, 103, 104, 108, 114, 116, 120, 122, 133] |
|  | Surveys | 14 (26%) | [80, 89, 91, 99, 105, 106, 109-111, 113, 117, 119, 121, 132] |
|  | Interviews/Focus Groups | 5 (9%) | [11, 88, 98, 112, 135] |
|  | Survey/questionnaires and Interviews | 13 (24%) | [8, 79, 81, 82, 85, 86, 90, 93, 96, 101, 102, 107, 118] |
|  | Others (Reflective journaling and Formal examinations results) | 3 (6%) | [115, 131, 134] |
| Mode of measurement | Likert scale | 20 (37%) | [80, 82, 87, 89, 94-97, 100, 102, 105, 107-111, 114, 116, 120, 122] |
|  | Close ended questions | 5 (9%) | [8, 36, 78, 91, 133] |
|  | Mixed approaches such as focus groups, telephone interview transcripts, yes/no questioning, free text and thematic analysis | 17 (32%) | [11, 79, 83-86, 88, 92, 93, 98, 104, 106, 112, 117-119, 121] |
|  | Not mentioned | 12 (22%) | [2, 81, 90, 99, 101, 103, 113, 115, 131, 132, 134, 135] |
| Points of evaluation | 1 point | 45 (84%) | [8, 11, 36, 78, 79, 81-84, 86-95, 97-106, 110-122, 132, 133, 135] |
|  | More than 1 point at fixed intervals | 5 (9%) | [2, 107-109, 131] |
|  | Continuous and after every meeting | 4 (7%) | [80, 85, 96, 134] |
| Validity of tools used for mentoring’s assessment | Unvalidated new tools | 42 (78%) | [8, 11, 36, 78, 79, 81-84, 86-99, 102-105, 107, 108, 110, 112-114, 116-122, 132, 133] |
|  | Modified from existing tools | 6 (11%) | [2, 80, 100, 106, 109, 111] |
|  | No mention of tool design | 6 (11%) | [85, 101, 115, 131, 134, 135] |
| Target of the tool’s assessment | Mentee | 29 (53%) | [2, 8, 11, 78, 81, 87, 92, 94, 95, 97-100, 102, 104-106, 109, 110, 113-117, 120, 121, 131, 134, 135] |
|  | Mentor | 10 (19%) | [79, 83, 84, 89-91, 111, 118, 119, 122] |
|  | Mentee and Mentor | 15 (28%) | [36, 80, 82, 85, 86, 88, 93, 96, 101, 103, 107, 108, 112, 132, 133] |
| Assessment setting | Medical school (involving medical students) | 22 (45%) | [11, 78, 80, 82, 84, 87, 89, 93, 95, 97, 100-102, 104, 107, 109, 116, 118-120, 134, 135] |
|  | University hospital and/or academic medical centre | 24 (49%) | [2, 8, 81, 83, 85, 88, 90, 94, 96, 99, 103, 105, 106, 110, 111, 113-115, 117, 121, 122, 131-133] |
|  | Regional/Multi-institutional (e.g. across NHS hospitals) | 3 (6%) | [79, 98, 112] |
