# Supplementary material for: Assessing mentoring: A scoping review of mentoring assessment tools in internal medicine between 1990 and 2019
Source: PLoS One. 2020 May 8;15(5):e0232511. doi: 10.1371/journal.pone.0232511 (PMC7209188; doi:10.1371/journal.pone.0232511)
Supplement: S2 Table — (DOCX) [file pone.0232511.s005.docx]

**S2 Table. Key points and summary of Domains of Mentoring Assessed and Assessment methods**

|  | Author and Year | The assessed | Method of assessment | Mentee-Mentor Relationship | The Mentor | Program related | Mentee Growth | Mentoring process | Validation |
| --- | --- | --- | --- | --- | --- | --- | --- | --- | --- |
| 1 | Ahmed et al 2017 | Mentee | Questionnaire | √ |  | √ |  | √ | X |
| 2 (abstract) | Borum et al 2015 | Both | Survey | √ |  |  |  |  | X |
| 3 (abstract) | Chandler et al 2015 | Mentees | Outcome measures/ Examination |  |  |  |  |  | Nil |
| 4 (abstract) | Chandler et al 2015 | Mentees | Survey |  |  | √ | √ |  | X |
| 5 | Connor et al 2000 | Mentor | Questionnaire + focus group | √ |  | √ | √ |  | X |
| 6 (abstract) | Devi et al 2010 | Mentee | Questionnaire |  |  |  | √ |  | X |
| 7 (abstract) | Dimitriadis et al 2012 | Both | Survey (Modified Mentorship Profile Questionnaire and Mentorship Effectiveness Scale) | √ | √ | √ |  | √ | Existing tool |
| 8 | Fornari et al 2014 | Mentor | Survey |  | √ | √ |  | √ | X |
| 9 | Frei et al 2010 | Both | Survey, Questionnaires, Interviews |  |  |  | √ |  | Nil |
| 10 | Ho et al 2017 | Mentee | Survey, Interviews |  |  | √ | √ |  | X |
| 11 | Herrera-Añazco et al 2015 | Mentee | Questionnaire |  |  |  | √ |  | X |
| 12 | Farrell et al 2012 | Mentee | Reflective journaling |  | √ |  |  |  | Nil |
| 13 | Ali et al 2015 | Mentee | Questionnaire (Modified from literature and the Mentorship Effectiveness Scale developed by the John Hopkins University School of Nursing) |  |  |  | √ | √ | Existing tool |
| 14 | Sadiq et al 2013 | Mentee | Questionnaire |  | √ |  |  | √ | X |
| 15 | Pololi et al 2016 | Mentor | Survey (Modified from the validated and reliable C-Change Faculty Survey) |  |  |  |  |  | Existing tool |
| 16 (abstract) | Moskowitz et al 2010 | Mentee | Survey |  |  | √ | √ |  | X |
| 17 | Rabatin et al 2004 | Both | Interview and audiotaped meetings | √ |  | √ |  | √ | Nil |
| 18 | Payer et al 1996 | Mentee | Survey |  | √ |  |  |  | X |
| 19 | Morrison et al 2014 | Mentee | Outcome measures/Years to promotion |  |  |  | √ |  | Nil |
| 20 | Selwa 2003 | Mentee | Survey | √ | √ |  | √ |  | X |
| 21 | Sheikh et al 2016 | Both | Questionnaire | √ |  |  |  |  | X |
| 22 | Ramanan et al 2006 | Mentee | Questionnaire | √ |  | √ |  | √ | X |
| 23 | Rehman et al 2014 | Mentee | Questionnaire |  |  |  | √ |  | X |
| 24 | Jackson et al 2003 | Mentee | Individual telephone interview + Close-ended questions | √ |  |  | √ |  | X |
| 25 | Levine et al 2013 | Mentee | Focus group of female medical students | √ | √ |  |  |  | X |
| 26 | Levy et al 2004 | Mentor | Questionnaire | √ | √ | √ |  | √ | X |
| 27 | Stenfors-Hayes et al 2010 | Mentor | Questionnaire + semi-structured interviews |  | √ |  |  |  | X |
| 28 | Sozio et al 2017 | Mentee | Questionnaire | √ |  | √ |  |  | X |
| 29 | Thorndyke et al 2006 | Both | 1) Multiple post-session evaluation  2) Mentee self-assessment before and after programme  3) Extensive post course survey  4) Mid-point survey  5) Mentor survey  6) Longitudinal study tracking career paths of participants |  |  | √ | √ |  | X |
| 30 | Kurre et al 2012 | Both | Survey and Interview |  |  |  |  | √ | X |
| 31 | Thorndyke et al 2008 | Both | 1) Pre- and post- program self-assessment  2) Mid-program survey of mentors and mentees  3) End-of-program questionnaire  4) Longitudinal surveys at regular intervals following program |  |  |  |  | √ | X |
| 32 | Kukreja et al 2017 | Both | Questionnaire + Focus group discussion | √ | √ | √ |  |  | X |
| 33 | Straus et al 2009 | Both | Semi-structured telephone interviews |  | √ | √ |  |  | X |
| 34 | Kalen et al 2015 | Mentee | Individual semi-structured interviews |  |  |  |  |  | X |
| 35 | Wasserstein et al 2007 | Mentee | Close-ended questions |  |  | √ |  |  | X |
| 36 | Steven et al 2008 | Both | Individual semi-structured interviews (face-to-face or interviews) |  |  |  | √ |  | X |
| 37 | Usmani et al 2011 | Mentors | Survey | √ | √ |  |  |  | X |
| 38 | Kirsling et al 1990 | Mentors - Senior faculty members | Interviews using questionnaires | √ |  |  |  | √ | X |
| 39 | Luckhaupt et al 2005 | Mentors | Questionnaire | √ |  | √ |  |  | X |
| 40 (abstract) | Miedzinski et al 2009 | Both | Questionnaire | √ |  | √ | √ |  | X |
| 41 | Meinel et al 2011 | Mentors | Questionnaire | √ | √ | √ |  | √ | X |
| 42 | Lin et al 2015 | Mentees - 5th year medical students | Survey (Modified Scandura and Ragins) |  |  |  | √ |  | Existing tool |
| 43 | Shollen et al 2014 | Mentee - Full time faculty | Survey (Adapted from John Hopkins University School of Medicine) |  |  |  | √ |  | Existing tool |
| 44 | Usmani et al 2016 | Mentees: students | Questionnaire | √ |  |  | √ | √ | X |
| 45 | Kalen et al 2012 | Mentees: students | Interviews |  |  |  |  |  | Nil |
| 46 | Stamm et al 2011 | Mentees - Graduates | Questionnaire (Mentoring Support Questionnaire, Objective and Subjective Career Success tools) | √ |  |  | √ |  | Existing tool |
| 47 (abstract) | Nelson et al 2014 | Mentee - Current faculty and fellows | Surveys | √ |  | √ | √ |  | X |
| 48 | Schäfer et al 2015 | Mentees | Questionnaire | √ | √ |  | √ |  | X |
| 49 | Iversen et al 2014 | Mentees | Questionnaire and Interviews | √ | √ | √ | √ |  | X |
| 50 | Heeneman et al 2019 | Both | Questionnaire |  | √ |  | √ |  | X |
| 51 | Elez et al 2019 | Mentors | Survey |  | √ | √ |  | √ | X |
| 52 | Ong et al 2018 | Mentees | Questionnaire and Examination Pass Rate |  |  | √ | √ | √ | X |
| 53 | Walensky et al 2018 | Both | Survey |  | √ |  | √ |  | X |
| 54 | Ogdie et al 2018 | Both | Focus group or interview and Survey | √ | √ | √ |  | √ | X |
